# Expanding the Focus of Occupational Safety and Health: Lessons from a Series of Linked Scientific Meetings

**DOI:** 10.3390/ijerph192215381

**Published:** 2022-11-21

**Authors:** Paul A. Schulte, George L. Delclos, Sarah A. Felknor, Jessica M. K. Streit, Michelle McDaniel, L. Casey Chosewood, Lee S. Newman, Faiyaz A. Bhojani, Rene Pana-Cryan, Naomi G. Swanson

**Affiliations:** 1 Advanced Technologies and Laboratories International, Inc., Gaithersburg, MD 20878, USA; 2 Southwest Center for Occupational and Environmental Health, The University of Texas Health Science Center at Houston School of Public Health, Houston, TX 77030, USA; 3 National Institute for Occupational Safety and Health, Atlanta, GA 30333, USA; 4 National Institute for Occupational Safety and Health, Cincinnati, OH 45226, USA; 5 Center for Health, Work & Environment and Department of Environmental and Occupational Health, Colorado School of Public Health, CU Anschutz, University of Colorado, Aurora, CO 80045, USA; 6 Shell USA, Inc., Houston, TX 77079, USA; 7 National Institute for Occupational Safety and Health, Washington, DC 20024, USA

**Keywords:** expanding occupational safety and health paradigm, future of work, occupational safety and health, transdisciplinary, inequality, training and education, research methods, psychosocial, mental health, well-being, socioeconomic, climate, pandemic, strategic foresight

## Abstract

There is widespread recognition that the world of work is changing, and agreement is growing that the occupational safety and health (OSH) field must change to contribute to the protection of workers now and in the future. Discourse on the evolution of OSH has been active for many decades, but formalized support of an expanded focus for OSH has greatly increased over the past 20 years. Development of approaches such as the National Institute for Occupational Safety and Health (NIOSH)’s Total Worker Health^®^ concept and the World Health Organization (WHO)’s Healthy Workplace Framework are concrete examples of how OSH can incorporate a new focus with a wider view. In 2019, NIOSH initiated a multi-year effort to explore an expanded focus for OSH. This paper is a report on the outputs of a three-year cooperative agreement between NIOSH and The University of Texas School of Public Health, which led to subject matter expert workshops in 2020 and an international conference of global interest groups in 2021. This article traces the background of these meetings and identifies and assesses the lessons learned. It also reviews ten thematic topics that emerged from the meetings: worker health inequalities; training new OSH professionals; future OSH research and practice; tools to measure well-being of workers; psychosocial hazards and adverse mental health effects; skilling, upskilling and improving job quality; socioeconomic influences; climate change; COVID-19 pandemic influences; and strategic foresight. Cross-cutting these themes is the need for systems and transdisciplinary thinking and operationalization of the concept of well-being to prepare the OSH field for the work of the future.

## 1. Introduction

The nature of work, the workforce and the workplace have changed and will change even more in coming years [1,2,3,4,5,6,7]. These changes will be profound, broadly impacting the world of work and creating new hazards and outcomes (Table 1). The predominant expected trend will be toward more adverse psychosocial effects of work, although some long-known chemical, physical, biological, and ergonomic hazards will persist and new ones may emerge [3,8,9,10]. Mental health problems such as depression, burnout, anxiety, and fatigue are already increasing rapidly and are expected to continue to grow, as will their costs to the global economy [11,12,13,14,15,16]. Along with demographic, social, and political changes, the impact of technology, climate change and globalization will also be significant [5]. In many ways, the COVID-19 pandemic has accelerated some of these trends and is likely to change work in the future, as will other pandemics [17].

The complex future interactions of how workers will be employed and the jobs they will perform will give rise to a broad range of new hazards, necessitating the occupational safety and health (OSH) field to expand its focus and evolve strategies that both protect workers and support enterprise productivity [1,2,3]. This overview of the world of work and proposal for an expanded focus for OSH was presented in a previous study, which is described below [18] and serves as the foundation for the current article and the cooperative agreement described herein.

**Figure 1:**
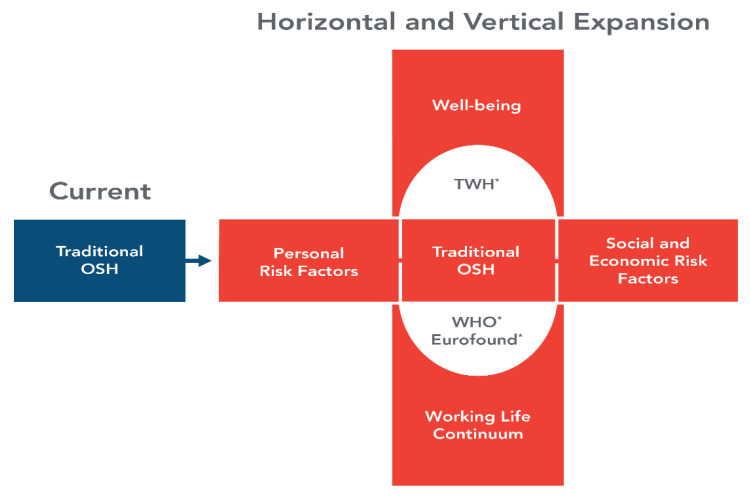
Model for an expanded focus for the occupational safety and health field [18,19].

Schulte et al. [18] proposed the expanded focus model presented in Figure 1 [18,19]. The model includes expansion in two directions: (1) horizontally, to include not only traditional OSH but also consideration of personal, social, and economic risk factors and (2) vertically, to foster a view of the entire working life and the use of an overarching concept of well-being to encompass the complexities of the changing world of work.

Calls for the OSH field to evolve are not new. A broad range of literature identifies the need and direction for OSH evolution (Table S1 and Figures S1–S4 in the Supplementary Materials) [2,3,7,8,9,10,18,19,20,21,22,23,24,25,26,27,28,29,30,31,32,33,34,35,36,37,38,39,40,41,42,43,44,45,46,47,48,49,50,51,52,53,54,55,56,57,58,59,60,61,62,63,64,65,66,67,68,69,70,71,72,73,74,75,76,77,78,79,80,81,82,83,84,85,86,87,88,89,90,91,92,93,94,95,96,97,98,99,100,101,102,103,104,105,106,107,108,109,110,111,112,113]. This history of an expanded focus for OSH, which is discussed in the Supplementary Materials, draws on literature that anticipates how OSH will be practiced in a future that is quite different from the past; involves conceptual, psychological, technical, and ethical expansions; and addresses only the modern eras (after 1970). Overall, the literature anticipates how future OSH will be practiced in a world of work quite different from the past. Novel skills, competencies, scope, research, and practice will be needed. Regardless of these changes, employer responsibility to provide safe and healthy work and governments to set limits remains a constant. The cooperative agreement described in this paper brought forth *Expanding Occupational Safety and Health: An International Conference* (Ex4OSH conference) and two subject matter expert workshops addressed themes that will pervade the future of OSH.

## 2. Materials and Methods

**Figure 2:**
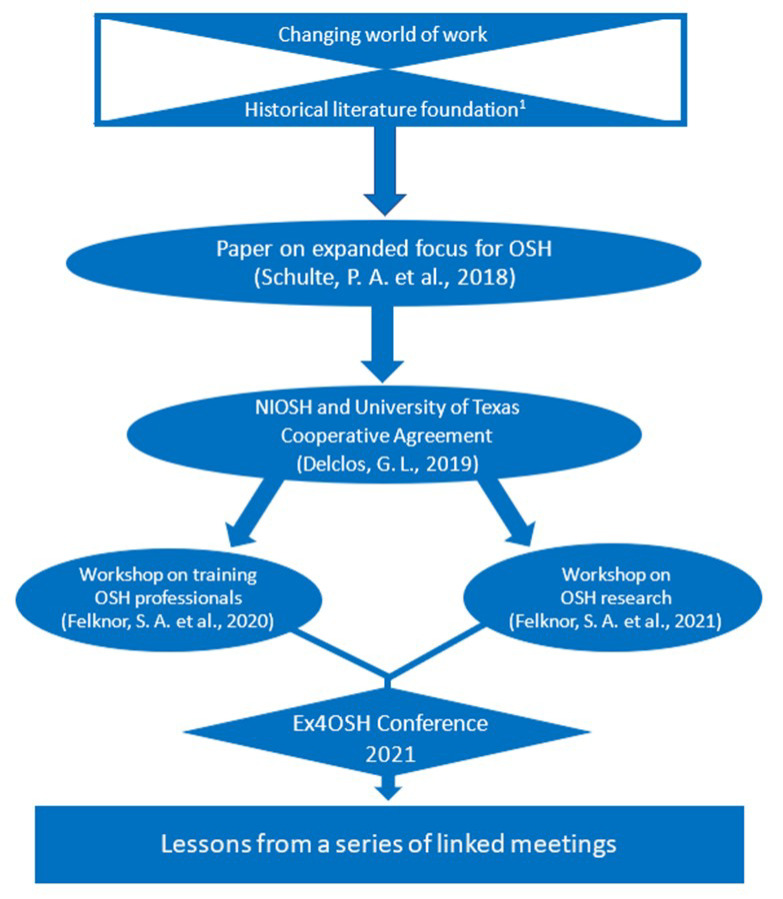
Overview of literature assessment and scientific meetings [18,19,76,114] (^1^ See Table S1 in Supplementary Materials for historical literature foundation).

This article builds on a horizon scan of the literature (Figures S1–S4 in the Supplementary Materials) to analyze the outputs of two 2020 subject matter expert workshops and a broad 2021 stakeholder-based international conference (Ex4OSH) (Figure 2) [18,19,76,114] (go.uth.edu/EX4OSH). These meetings were a product of a 2019 3-year cooperative agreement, entitled *Shaping the future to ensure worker health and well-being: shifting paradigms for research, training, and policy*, between the National Institute for Occupational Safety and Health (NIOSH) and The University of Texas Health Science Center at Houston School of Public Health, Southwest Center for Occupational and Environmental Health [114]. The cooperative agreement application was supported by a broad range of interest groups from labor, business, academia, and government.

The section below synthesizes the key themes, recommendations, and next steps from all three meetings. The recommendations from the two preliminary workshops were developed by a group consensus process. These outputs were then used in the planning of the Ex4OSH conference. The authors of this article drew on the Ex4OSH outputs and presentations to identify emergent themes and make observations and recommendations that might be useful in guiding the field in the future.

## 3. Significant Emergent Themes from the Workshops and Ex4OSH Conference

Worker well-being and the need for OSH to expand and evolve were the central themes of all three meetings, which also considered how this should drive changes in approaches to training, research, and policymaking. OSH should position itself to lead efforts to prevent workplace hazards, keep workers safe and contribute to overall worker well-being [80,115,116,117]. The problems of current and future work—including precariousness, lack of meaning, unhealthy organizational conditions, low wages, non-standard work arrangements, changing workforce demographics, skills mismatch and deficiency, powerlessness, overload, and others—require the application of a broad overlying concept of well-being, which implies a more holistic approach to the status of workers in terms of health, satisfaction, and flourishing [5,7,118,119]. For well-being to be more than a conceptual goal, it should be operationalized [48,63,119] (see Section 3.4, on tools). Several approaches to operationalizing well-being have been launched, including the Centers for Disease Control and Prevention (CDC)/NIOSH Total Worker Health^®^ approach [74] and the International Labour Organization (ILO) focus on decent work [75], which is one of the 2030 sustainable development goals (SDG-8). Decent work “involves opportunities for work that is productive and delivers a fair income, security in the workplace and social protection for families, better prospects for personal development and social integration, freedom for people to express their concerns, organize and participate in decisions that affect their lives and equality of opportunity and treatment for all women and men” [75]. Both work and non-work factors reciprocally affect the health and well-being of workers [120]. Risk factors in the workplace can affect or be affected by other factors long considered as non-work-related [121,122,123]. For example, data were presented at Ex4OSH that obesity, cigarette smoking, cardiovascular disease, mental health conditions and many other outcomes were strongly related to work conditions, particularly “iso-strain” (constant strain), which is related to low-control and high-demand types of work [89,124,125,126,127,128]. Extensive data show that workers are suffering from stress, depression, anxiety, burnout, and other adverse mental health effects [90,118,129,130]. These outcomes should be studied over a working lifetime to fully capture their magnitude, impact, and responsible factors [27,131].

To address current and future well-being and the hazards and risks that workers face, today’s paradigm must shift from the biomedical model to the more expansive biopsychosocial model [59,86,132,133,134]. The biomedical model served OSH well but is limited and reductionist, addressing hazards one at a time, in part because of regulatory and structural drivers. That approach will not suffice in the current or future work environment, where more complex issues of change management, job quality, stress, equity and worsening mental health among workers are pertinent.

Refocusing on well-being and decent work under a biopsychosocial model has many implications for OSH: training professionals, conducting research, and developing policy directions and dimensions; and growing the field. In the wake of the COVID-19 pandemic, the changes in the world of work are accelerating, highlighting the urgency to keep pace by similarly evolving worker health, safety, and well-being efforts [77]. The following subsections focus on ten emergent themes from the conference and workshops: worker inequalities; training new OSH professionals; future OSH research and practice; tools to measure well-being of workers; psychosocial hazards and adverse mental health effects; skilling, upskilling and improving job quality; socioeconomic influences; climate change; influence of the COVID pandemic on OSH; and strategic foresight. A summary of key issues and recommendations for each theme is included in Table 2.

### 3.1. Worker Health Inequalities

As noted in the Ex4OSH opening address by the U.S. Secretary of Labor, Marty Walsh, a safe and healthy workplace should never be the privilege of a few, and it is every worker’s right to return home safe and sound at the end of a work shift [135]. The COVID-19 pandemic represents an opportunity to underscore the need to make broad strides in OSH to address worker health inequalities [17]. For example, during the pandemic, not all workers were able to transition to work from home or remotely. The health impacts fell disproportionately on essential workers and their families, and the economic impacts fell heavily on low-wage workers, women, racial minorities, and immigrants [136,137,138]. There was clear proof that workforce disparities are unjust and unsustainable [136]. However, even before the pandemic, growing disparities between the amount of resource going to capital and the amount going to labor were widely acknowledged [139]. From 1980 to 2019, annualized earnings growth in the United States averaged approximately 1% for a vast number of workers [135]. In contrast, those at or above the 95th percentile of income distribution saw their annual earnings growth increase rapidly, from 1.5% to 6% [60,135,140,141,142]. This earnings inequality is driven in part by organizational restructuring, the fissured workplace and increasing inequality between companies [143].

While work has long been acknowledged as a social determinant of health, the assessment of its role has generally been limited to models of workplace physical, chemical, ergonomic, and safety exposures [85,144,145]. However, the influence a job has on health goes well beyond specific physical working conditions, circumscribing workers’ lives and those of their families. Jobs determine how much money a worker has, the neighborhood they live in, the schools their children attend, as well as their community and political participation [145,146,147]. Emotionally, a job influences self-esteem and stress. Socially, a job impacts other factors such as status, connectedness, free time, and peer interactions and supports. In short, work is not only a social determinant of health in and of itself; it also significantly impacts and is impacted by other social determinants of health and well-being [85,86]. Nonetheless, it remains largely absent in health inequalities research [116,145], as do the roles of unemployment and underemployment [81].

Clearly, the distribution of work-related benefits and risks is a result of how society is structured along social axes (race/ethnicity, class, gender, nativity, etc.), how business is structured (competitive bidding, business size, subcontracting practices) and how jobs are structured (employment arrangements, shiftwork, autonomy, precarious work) [145]. The field of OSH needs to understand how structural disadvantages materialize and influence worker health [145]. The use of biopsychosocial model–focused research will be critical in this regard [145].

### 3.2. Training New OSH Professionals

The need to reorient OSH professional training with the future of work in mind was the main theme of the first preparatory workshop (February 2020) and a major focus of the Ex4OSH conference [76]. There was broad agreement that future OSH professionals should take a more holistic approach and be proactive in anticipating hazards in the future of work. Preparation will involve training in systems science and transdisciplinary approaches. Systems science involves understanding the interconnections of elements (systems) that are organized to achieve a specific purpose [76,148,149]. These methodologies are “thought to enable researchers and decision-makers to examine system components and the dynamic relationships between them at multiple levels from cell to society” [149]. A transdisciplinary approach is an “… integrative process that synthesizes and extends disciplinary-specific theories, concepts, or methods to create new models and language to address OSH issues [18].” These methods cross multiple disciplines and professions, resulting in a broader and more holistic approach to problem-solving strategies [59,76,150].

A critical issue will be incorporating systems science and transdisciplinary approaches into OSH training without overburdening an already demanding curriculum.

One possible approach is to train OSH professionals to be aware of, liaise with and work transdisciplinarily with related fields. At the least, collaboration on OSH topics should be encouraged between different disciplines. Another approach is to incorporate this new thinking into existing courses through individual or group projects and exercises that address Total Worker Health issues and global worker well-being [77]. Key content areas that might be addressed in these training activities are shown in Table 3 [151,152].

### 3.3. Future OSH Research and Practice

The second preparatory workshop examined how the future of work will impact OSH research and professional practice [19]. OSH researchers and practitioners will face new and exacerbated challenges in the future: new forms of work organization and technologies; continued erosion of the line between work and nonwork; electronic monotony; lack of organizational commitment to safety; emotional labor; digital surveillance; and expansion of psychosocial strain in many disease processes (“diseases of despair”) and work hazards in the context of socioeconomic, racial and gender disparities [153,154].

To address these challenges in OSH research, it is important to identify the pertinent affected parties and collaborators and examine ways to incorporate them into the discussion. The use of participatory research methods, which involves listening to workers, learning about their lived experiences, and then partnering with them to document problems and envision changes, has a rich history [155,156,157,158,159]. There are established participatory research methods engaging workers as partners to design effective and feasible solutions, although conducting and evaluating such research can be difficult [128]. Realistic evaluation, which is frequently used in public health research and in approaches such as Contribution and Impact Analysis, may be required [160,161].

The OSH field will also need to identify evidence-based approaches likely to move others—particularly employers—to action that benefits workforce health and safety. One useful approach may be documenting costs to employers or society to fix a problem [160,161]. Another example is showing the positive effects of high-quality jobs and employee involvement on organizational performance and employee well-being [162,163]. Additional research on the interaction of working conditions with broader economic and political issues (such as housing and transportation) will also be needed [89].

Addressing the principles of well-being and decent work in future OSH research will face important obstacles, such as those illustrated by the Healthy Work Campaign [164]. These include: (1) lack of consensus on a definition for “healthy work”; (2) lack of consensus on the role of work stressors in ill health; (3) ideological beliefs about health and illness; (4) economic barriers to fixing unhealthy working conditions; (5) inadequate tools to diagnose sick and unhealthy workplaces; (6) lack of fully-tested, workable interventions and implementation strategies for workplace change; and (7) lack of enforceable standards to ensure that work is safe and promotes health. Additionally, the argument has been made that “the shortfall in ‘good jobs’ can be viewed as a massive market failure—a kind of gross economic malfunction and not just a source of inequality and economic exclusion [165].” These obstacles can be overcome in part with innovative research approaches, engaging different allies and partners, especially those outside the usual framework, particularly intermediary organizations [166,167,168,169,170,171].

### 3.4. Tools to Measure Well-Being of Workers

Operationalizing and measuring well-being were key issues raised in the Ex4OSH conference. Two tools that were presented were the Thriving from Work Questionnaire and the Worker Well-Being Questionnaire (WellBQ).

#### 3.4.1. Thriving from Work Questionnaire

Thriving in a work environment has been described as “a state of positive mental, physical and social functioning in which workers’ experiences of their work and working conditions enable them to thrive in their overall lives, contributing to their ability to achieve the full potential in their work hours and community” [172]. The Thriving Framework Questionnaire is a validated 87-item survey that has both a long and short form. It is intended for use across working populations or organizations; in periodic surveillance across a worker population or within an organization; or as an organizational diagnostic tool to identify priority areas for interventions to improve worker well-being [172]. Currently the investigators are examining thriving across various multiple work settings. One ongoing study, for example, is using the tool to identify relationships of working conditions (such as supervisor support, safety climate, or scheduling) and workplace policies and practices with workers’ thriving from work [172].

#### 3.4.2. NIOSH Worker Well-Being Questionnaire

There is no widely accepted definition of well-being. Some instruments capture aspects of it, but none capture the totality of well-being related to work. In this regard, the NIOSH WellBQ (available at https://www.cdc.gov/niosh/twh/wellbq/default.html (accessed on 11 April 2022) represents an important step forward. This 68-item questionnaire was recently released for public use, after testing and validation. It is based on a framework that considers well-being as an integrative concept that characterizes the quality of life with respect to an individual’s health and work and related environmental, organizational, and psychosocial factors. It also examines the experience of positive perceptions and presence of constructive conditions at work and in other areas of life that enable workers to thrive and achieve their full potential [119].

### 3.5. Psychosocial Hazards and Adverse Mental Health Effects

The literature is rich on psychosocial hazards and the risks related to future changes in work [1,2,3,14,16,18,78,80,90,99,113,117,129,170,171,172,173,174,175]. Evidence of the increasing prevalence and incidence of adverse mental health effects and their costs to workers and organizations is also growing [5,11,113,176,177,178]. These effects include anxiety, depression, loneliness, suicidal ideation, psychological strain, and related physical conditions, including cardiovascular diseases, musculoskeletal disorders, and other systemic disorders. Although these hazards and effects predate the COVID-19 pandemic, they were accelerated by it.

The conditions of work can adversely influence psychological responses, particularly when there are high demands and low control or limited latitude for decision-making [162]. Lacking a sense of purpose can also be a factor that, alone or combined with work conditions, leads to adverse physical and mental effects [11,100,164,177,179].

A key theme highlighted in the workshops and Ex4OSH is the awareness of increasing prevalence of psychosocial hazards and mental health disorders related to work [17,18,129,179]. There is a growing number of psychosocial hazards brought about by new forms of work organization; precarious employment; stress; exploitation; migration; irregular schedules; low decision latitude; lack of organizational commitment to safety; absence of a strong social contract; and work hazards in the context of socioeconomic, racial and gender disparities and demographics [10,80,99,113,125,178,180,181,182,183,184,185,186].

While there is some guidance in various countries on assessing and preventing adverse effects from psychosocial hazards this is still a relatively new area for OSH [16,173,187]. Some countries do have standalone guidance. Canada, for example, initiated *Psychological Health in the Workplace* in 2013 [188] and New Zealand initiated *Psychosocial hazards in work environments and effective approaches for managing them* in 2019 [90]. One approach that could have broad applicability is the International Standards Organization (ISO) 45003, *Occupational health and safety management—Psychological health and safety at work: guidelines for managing psychosocial risks*, which represents the most comprehensive global effort to address psychosocial hazards and is intended for use in conjunction with ISO 45001 (*Occupational health and safety management systems*) [189]. Another strong approach to addressing psychosocial hazards is the development of a psychosocial safety climate [190,191]. Psychosocial safety climate refers to “a climate for psychological health and safety in workers,” reflects the balance of concern by management about psychological health versus productivity and is an objective in building a psychologically productive workplace [54,190].

### 3.6. Skilling, Upskilling and Improving Job Quality

Much of the concern about the future of work is that, owing to technological advances, workers may not be able to obtain or keep jobs because of inadequate skills [3,4,5]. Many jobs of the future also do not currently exist, making preparation and transitioning uncertain [4,192]. Such skill-related issues affect both white-collar and blue-collar workers and generally arise because of technological changes [193]. White collar workers fear job loss due to replacement by artificial intelligence, algorithms, and robots. Skills mismatch and development and skills transfer issues affect blue collar workers in skilled trades. One interesting approach presented at Ex4OSH to this dilemma is the “union-construction model” [194], built on the foundation of peer-to-peer training and the apprenticeship program. This model has had wide success in the United States and apprentice programs are effective worldwide [195].

The limitations workers face relate not only to evolving personal skills and competencies but also to job quality and wages [196]. In the United States in 2017, 44% of working adults, aged 26–64 years, earned $15/h or less and these disproportionately were women or racial minorities [196]. While there are beneficial effects of the standard approaches to job quality—regulation, strengthening countervailing power of workers and skill framing—the efficiency of these could be enhanced by using an industry or sector strategy [196]. Improving job quality can improve both the work experience and organizational productivity and therefore deserves greater emphasis from the OSH community.

### 3.7. Soceioeconomic Influences

Future OSH hazards will be increasingly influenced by socioeconomic factors such as business organization, wages, industry specific public processes, insurance, unemployment, and efforts to bring about decent work [27,34,41,52,55,60,80,81,87,197,198,199,200,201,202,203]. Although there has not been a systematic assessment of the role of macroeconomic factors on occupational morbidity, mortality and injury, there is literature that identifies individual socioeconomic factors. Increasing GDP, for example, has been related to decreasing occupational injuries in Austria, although no direct causal linkage could be ascertained and generalization to other countries is limited [204].

There is also evidence supporting a link between job and income insecurity and negative health outcomes [187,201,202,205]. A critical means to address workplace hazard and adverse effects involves placing a systematic focus on organizational culture and climate [54,185,190,192,206]. Changing organizational design and work arrangements such as platform work are linked with job flexibility and have implications for work-life. This may be a growing issue in the future [82]. To cope with this, stricter government policy and enforcement as well as agreements between employers’ organizations and trade unions may be needed.

### 3.8. Climate Change

Climate change, more severe than in the past, will influence both OSH training and research. *The Lancet* has argued that “climate change is the biggest global threat of the 21st century and tackling climate change could be the greatest global opportunity of the 21st century” [207]. Climate change overlaps many of the other emerging themes. Workers are one of the first groups to be adversely affected by climate change, reflected in greater and more serious exposures, and they manifest health effects earlier than the general population [50,208,209,210,211,212], akin to being “climate canaries” [211]. Climate-related worker hazards include increased ambient temperature; air pollution; ultraviolet radiation exposure; extreme weather; vector-borne diseases and expanded habitats; industrial transitions and emerging industries; and changes in the built environment [209]. In addition to the many physical effects identified, adverse mental health effects from climate change are predicted to grow extensively in the future [207,210]. The distribution of the deleterious effects of climate-related factors not only occurs by occupation and industry but in conjunction with social and economic inequalities. Consequently, it will be essential to include climate-related knowledge in OSH curricula and address climate-related hazards in research and policy development.

### 3.9. COVID-19 Pandemic Influences on OSH

The COVID-19 pandemic is an historic moment in human history, with a large impact on work. All workers around the world have been affected by the pandemic [213]. Broadly speaking, the entire population has faced new psychosocial risks, but a comprehensive approach to these risks is lacking. In response, OSH practitioners should make the link between occupational health and public health evident [80,213,214]. Unfortunately, it is often difficult to understand or prevent psychosocial risks and hazards because they are not necessarily tangible, sometimes emerging only when their impact is seen [16]. The ILO, however, has presented a holistic approach that can address the broad range of psychosocial factors in the world of work [214].

Prior to the pandemic, worker well-being was already decreasing in many sectors [118,164,214,215,216]. The pandemic further influenced this decline by accelerating the realization of future predictions for the well-being of the workforce [18,122,215]. Most investigators could not access workers at worksites to conduct intervention research [217,218,219]. The pandemic illustrated the utility of Total Worker Health, an integrated approach that avoids the traditional siloes of OSH by focusing comprehensively on the whole worker [219]. Total Worker Health may advance worker equity by encouraging employers to look beyond work and consider how they can help workers on broader issues, especially during times of public crises (e.g., pandemics).

### 3.10. Strategic Foresight

Given the speed of change facing OSH and growth in challenges and uncertainties, new approaches to preparing for the future are paramount. Strategic foresight is an action-oriented planning discipline that can help test current strategy and create transformative change. Strategic foresight recognizes that the future is not predetermined or predictable, and it is designed to ask questions such as what may be coming, how it might affect us, and what we can do today to move toward a desired future [151]. The practice of strategic foresight involves both mapping, or creating visions of, the future and assessing the implications and critical issues associated with those future views [151,220]. Together, these activities can then reduce the likelihood that we are unprepared for future change. The insights gained from strategic foresight can be used as a complement to, rather than a substitute for, traditional strategic planning that relies heavily on historic data and trends [151]. The number of organizations using strategic foresight to create and explore plausible work future is growing worldwide [151].

Despite its popularity as an input to many different types of business and decision-making efforts, strategic foresight has been underutilized in OSH. To help bring the practice to OSH, NIOSH has developed a tailored approach to strategic foresight that supports the expanded focus [151]. The NIOSH Foresight Framework for OSH builds on the work of Bishop and Hines [220,221] and involves six interrelated stages: framing the OSH domain; scanning for signals of change; developing plausible future scenarios; exploring implications and options of plausible futures; designing strategies to prepare for and influence the future; and monitoring for new signals of change [151,152,222]. Well-known OSH resources, such as the NIOSH TWH program’s list of extensive issues related to enhancing working well-being, can help to focus future foresight efforts within a meaningful OSH domain [151,152]. To ensure that strategic foresight can be sustained in OSH, it will be necessary to build capacity within the OSH community of research to practice. The Foresight Competency Model developed by the Association of Professional Futurists can serve as a guide for developing knowledge, skills, and abilities within OSH [151,152,220].

## 4. Conclusions

The workshops and conference meetings described in this article provided a view of an expanded focus for the OSH field that considers the cumulative and compounding effects of a broad list of factors and the interactions they have on worker health outcomes, particularly mental health outcomes and well-being [13,25,80,90,152]. However, understanding and proactively managing the ever-growing and evolving list of risk factors and outcomes requires us to expand not only how we think about OSH but the urgency with which we act to address these changes.

The meetings added depth to discussion of a model for an expanded focus for OSH (Figure 1), which was first published in 2019 and calls for the expansion from traditional OSH focus to one that includes consideration of the impact of personal and socioeconomic factors over the working lifetime, aimed at achieving worker and workforce well-being. Well-being has long been considered in OSH but often merely as a descriptive aspiration in a conjunctive phrase (“…and well-being”) rather than as a measurable variable in research or a goal, target, or indicator in policy and practice. The meetings also examined the operationalization and use of the concept of well-being in OSH, setting the stage for evolution of the field to address complex and difficult issues in the work of the future.

Recommendations from the first two subject matter expert workshops [19,76] focused on how the future of work will shape the OSH professional and research and practice in the future. Critical in training new OSH professionals will be the incorporation of systems thinking, skill in negotiations and understanding power relations, transdisciplinarity and foresight approaches into the curricula. New training methods and approaches will be needed to meet the demands of an increasingly mobile and distributed workforce, such as rapid-readiness training. In an expanded focus for OSH, future research and practice will require a broader view of complex issues and transdisciplinary paradigms to better prepare for new models of employment. The Ex4OSH conference reiterated the need to incorporate well-being as an overarching construct for OSH research, practice and policy. Tools such as the NIOSH WellBQ and Thriving from Work Questionnaire provide a systematic approach to operationalizing these constructs.

A major driver of well-being is the prevention and control of adverse effects from psychological hazards. The OSH field will need to be adept at compliance and implementation of innovative psychological health standards such as in Canada (CAN/CSA–Z1002–13/BNQ4700-803/2013) [188] and the ISO 45003 standards [189].

The impact of climate change on the quality of work and on mental health will also be more pronounced [196,210]. The growing occupational impacts of climate-related hazards still lack adequate investment in research, surveillance, and risk management.

If the OSH field is to address the new complexities of the future, it will need to have a paradigm that supports an expanded focus. The biopsychosocial [86,145] and Total Worker Health [74] paradigms can serve as a foundation on which the OSH field can position itself to have such an expanded focus that more clearly envisions well-being for workers.

There are many practical, legal, and social considerations when using the concept of well-being in occupational safety and health. Key issues pertain to worker privacy on factors that may influence the line between worker and employee responsibilities; costs related to well-being interventions; absence of a clear legal framework; and concern that addressing well-being will dilute scarce resources that would be needed for traditional OSH hazards [223]. Nonetheless, these challenges are surmountable, and data are increasing to link well-being with worker, enterprise, and national productivity. This may motivate broader adoption of the concept.

The meetings described herein represent an effort to ensure that the field of OSH looks forward so that it continues to be relevant and impactful and is more valuable to society because it takes a broader view of the issues affecting workers and the workforce. Clearly, the topics and speakers of these meetings reflect a selective process by the authors and to some extent their points of view. This article captures the major themes of the Ex4OSH conference and workshops: worker health inequalities; training new OSH professionals; future OSH research and practice; tools to measure well-being of workers; psychosocial hazards and adverse mental health effects; skilling, upskilling and improving job quality; socioeconomic influences; climate change; COVID-19 pandemic influences on OSH; and strategic foresight. Attention to these themes will be critical if the OSH field is to be effective in the future. Ultimately, these themes are generally conceptual as were most of the meetings. There is need for distilling the themes to practical outputs and guidance that is beyond the scope of this paper, but necessary if there is to be an expanded focus for the OSH field. Moreover, the effectiveness of the OSH field depends not only on expertise and research but at least as much on legislation and strategies to convince and, if necessary, require organizations to take specific actions.

## Figures and Tables

**Table 1:** The world of work is changing.

Work	Workforce	Workplace
Mosaic of old and new hazards Shift from physical to mental work More service work Work intensification Many jobs in a working lifetime Emotional labor	Greater distribution of older workers More immigrants More women Less unionization Increased chronic disease burden	New work arrangements More telecommuting Contractors and temporary workers More small businesses Decrease in social protection Pandemics Increased climate-related hazards

**Table 2:** Summary of Key Issues and Recommendations for Identified Themes from the Ex4OSH conference ^1^.

Theme	Key Issues	Recommendations
Worker health inequalities	Influence of a job goes beyond working conditions to circumscribing workers’ lives Disproportionate burden on essential workers from COVID-19 Growing disparities from resources going to capital vs. labor investments	Explore how structural disadvantages influence worker health Use a biopsychosocial model to focus research and address inequalities
Training new OSH professionals	Need to reorient OSH professional training to be mindful of the future of work	Take a more holistic approach to OSH that proactively anticipates hazards Utilize system science and transdisciplinary approaches
Future of OSH research and practice	OSH researchers will face new challenges that will need investigation Enhanced practices of OSH to address decent work and well-being will face important obstacles	Identify pertinent affected partners to participate in research discussions Consider a broad range of social, economic, political, and cultural factors in OSH research and practice
Tools to measure well-being of workers	Thriving from work Questionnaire NIOSH Well BQ questionnaire	Operationalize well-being
Psychosocial hazards and adverse mental health effects	Increased prevalence and incidence of adverse mental health effects from work New psychosocial hazards to work Trends exacerbated by COVID-19 The need for psychosocial safety climate	Develop and implement guidance for assessing and preventing work-related psychosocial hazards
Skilling, upskilling and improving job quality	Technological displacement of workers Skill-related issues for white- and blue-collar workers Utility of union construction approach	Standard approaches to job quality can be enhanced by using an industry of sector strategy
Socioeconomic influences	A variety of socioeconomic factors will influence future occupational hazards	Development and enforcement of stricter socioeconomic policies Agreement between employers and trade unions on work organization
Climate change	Biggest global threat of 21st century Workers are among the first to be adversely affected Seven categories of climate-related hazards	Include climate-related knowledge in OSH curricula
COVID-19 pandemic influences on OSH	Workers have been significantly affected by the COVID-19 pandemic Pandemic accelerated decline of well-being More awareness of psychosocial hazards Pandemic illustrated the utility of Total Worker Health	Strengthen the link between occupational health and public health Learn how to address the OSH effects of pandemics and sudden disruptions in timely and innovative ways

^1^ Recommendations from the Ex4OSH conference are based on guidance from the speakers and authors rather than consensus. Recommendations from the two pre-conference workshops are published elsewhere [19,76].

**Table 3:** Key Content Areas for Training OSH Professionals [152].

Content Area	Examples
New Investigation Strategies	Biopsychosocial model of OSH Systems thinking Futures thinking
Technology	Digitalization Societal reliance on technology Human-machine interface
Organizational Development	Organizational change Change management
Data Techniques	Data collection Data management Data analysis Data interpretation
Interpersonal skills	Social skills Communication Emotional intelligence Transdisciplinary teamwork

## Data Availability

Not applicable.

## Supplementary Materials

The following supporting information can be downloaded at: https://www.mdpi.com/article/10.3390/ijerph192215381/s1, Historical influences on an expanded focus for OSH; Table S1: Selected history of contributions to an expanding focus for occupational safety and health; Figure S1: Literature indicating conceptual expansion of OSH; Figure S2: Literature indicating psychological expansion of OSH; Figure S3: Literature indicating technical expansion of OSH; Figure S4: Literature indicating ethical expansion of OSH

## References

[1] Daheim C., Winterman O. (2016). 2050: The Future of Work. Findings of an International Study of the Millennium Project.

[2] Peckham T.K., Bker M.G., Camp J.E., Kaufman J.D., Seixas N.S. (2017). Creating the future for occupational health. Ann. Work. Expo. Health.

[3] Schulte P.A., Streit J.M.K., Sheriff F., Delclos G., Felknor S.A., Tamers S.L., Fendinger D., Grosch J., Sala R. (2020). Potential scenarios and hazards in the work of the future: A systemic review of the peer-reviewed literature and gray literature. Ann. Work. Expo. Health.

[4] The World Bank The World Development Report (WDR) 2019. *The Changing Nature of Work*. https://www.worldbank.org/en/publication/wdr2019.

[5] Balliester T., Elsheikhia A. (2018). The Future of Work: A Literature Review.

[6] Manyika J., Lund S., Jacques C., Bughin J., Woetzel J., Batra P., Ko R., Sanghvi S. (2017). What the Future of Work Will Mean for Jobs, Skills and Wages: Jobs Lost, Jobs Gained.

[7] Howard J. (2017). Nonstandard work arrangements and worker health and safety. Am. J. Ind. Med..

[8] Cox T., Griffiths A., Rial-Gonzalez E. (2000). Research on Work Related Stress.

[9] Coggon D. (2005). Occupational medicine at a turning point. Occup. Environ. Med..

[10] Leka S., Jain A. (2010). Health Impact of Psychosocial Hazards at Work: An Overview.

[11] Goetzel R.Z., Roemer E.C., Holingue C., Fallin M.D., McCleary K., Eaton W., Agnew J., Azocar F., Ballard D., Bartlett J. (2018). Mental health in the workplace: A call to action; proceedings from the mental health in the workplace. Public health summit. J. Occup. Environ. Med..

[12] Roehring C. (2016). Mental disorder top the list of most costly conditions in the United States: $201 Billion. Health Aff..

[13] Leka S., Jain A., Lerouge L., Lerouge L. (2017). Work-related Psychosocial risks: Key definitions and an overview of the policy context in Europe. Psychosocial Risks in Labour and Social Security Law: A Comparative Legal Overview from Europe, North America, Australia and Japan.

[14] Mykletun A., Harvey S.B. (2012). Prevention of mental disorders: A new era for workplace mental health. Occup. Environ. Med..

[15] Trautmann S., Rehm J., Wittchen H.-U. (2016). The economic costs of mental disorders. EMBO Rep..

[16] Jespersen A.H., Hasle P., Nielsen K.T. (2016). The wicked character of psychosocial risks: Implications for regulation. Nord. J. Work. Life Stud..

[17] Sigahi T.F.A.C., Kawasaki B.C., Bolis I., Morioka S.N. (2021). A systematic review of the impacts of Covid-19 on work: Contributions and a path forward from the perspectives of ergonomics and psychodynamics of work. Hum. Factors. Ergon. Manuf..

[18] Schulte P.A., Delclos G., Felknor S.A., Chosewood L.C. (2019). Toward an expanded focus for occupational safety and health: A commentary. Int. J. Environ. Res. Public Health.

[19] Felknor S.A., Streit J.M.H., McDaniel M., Schulte P.A., Chosewood L.C., Delclos G.L. (2021). How will the future of work shape OSH research and practice? A workshop summary. Int. J. Environ. Res. Public Health.

[20] Ferguson D., Brebner J. (1977). The psychologist and occupational health. Proceedings of the Annual Conference, Ergonomics Society of Australia and New Zealand.

[21] Karasek R.A. (1979). Job Demands, Job Decision Latitude, and Mental Strain: Implications for Job Redesign. Admin. Sci. Q..

[22] El Batawi M.A. (1984). Work-related diseases: A new program of the World Health Organization. Scand. J. Work Environ. Health.

[23] Samuels S.W. (1986). The Environment of the Workplace and Human Values.

[24] Antonovsky A., Cooper C.C., Kalima R., El-Batauri M. (1987). Health promoting factors at work: The sense of coherence. Psychosocial Factors at Work and Their Relation to Health.

[25] Sauter S.L., Murphy L.R., Hurrell J.J. (1990). Prevention of work-related psychological disorders: A national strategy proposed by the National Institute for Occupational Safety and Health (NIOSH). Am. Psychol..

[26] Ilmarinen J., Tuomi K., Eskelinen L., Nygard C.H., Huuhtanen P., Klockars M. (1991). Background and objectives of the Finnish research project on aging workers in municipal occupations. Scand. J. Work Environ. Health.

[27] Amick B.C., McDonough P., Chang H., Rogers W., Pieper C., Duncan G. (2002). Relationship between all-cause mortality and cumulative life course psychosocial and physical exposure, in the United States labor market 1968 to 1992. Psychosom. Med..

[28] Israel B.A., Schurman S.J., Hugentobler M.K. (1992). Conducting Action Research: Relationships between Organization Members and Researchers. J. Appl. Behav. Sci..

[29] Heaney C.A., Price R.H., Rafferty J. (1995). Increasing coping resources at work: A field experiment to increase social support, improve work team functioning, and enhance employee mental health. J. Organ. Behav..

[30] Sorensen G., Himmelstein J.S., Hunt M.K., Youngstrom R., Hebert J.R., Hammond S.K., Palombo R., Stoddard A., Ockene J.K. (1995). A Model for Worksite Cancer Prevention: Integration of Health Protection and Health Promotion in the WellWorks Project. Am. J. Heal. Promot..

[31] Quick J.C. (1996). Editorial. J. Occup. Health. Psychol..

[32] Siegrist J. (1996). Adverse health effects of high-effort/low reward conditions. J. Occup. Psychol..

[33] Adkins J.A. (1999). Promoting organizational health: The evolving practices of occupational health psychology. Prof. Psychol. Res. Pract..

[34] Dembe A. (1999). Social inequalities in occupational health and health-care for work-related injuries and illnesses. Int. J. Law Psychiatry.

[35] Rantanen J. (1999). Challenges for occupational health from work in the information society. Am. J. Ind. Med. Suppl..

[36] London L., Kisting S. (2002). Ethical concerns in international occupational health. Occup. Med..

[37] U.S. EPA (2003). Framework for Cumulative Risk Assessment.

[38] Lavis J.N., Robertson D., Woodside J.M., McLeod C.B., Abelson J. (2003). How can research organizations more effectively transfer research knowledge to decision makers?. Milbank Q..

[39] Quinn M. (2003). Occupational health, public health, worker health. Am. J. Public Health.

[40] Putnam K., McKibbin L., Wachs J.E. (2004). Managing workplace depression: An untapped opportunity for occupational health professionals. AAOHN J..

[41] Caruso C.C., Bushnell T., Eggerth D., Heitmann A., Kojola B., Newman K., Rosa R.R., Sauter S.L., Vila B. (2006). Long working hours safety and health: Toward a national research agenda. Am. J. Ind. Med..

[42] Schulte P.A. (2006). Emerging issues in occupational safety and health. Int. J. Occup. Environ. Health.

[43] Bakker A.B., Demerouti E. (2007). The Job Demands-Resources model: State of the art. J. Manag. Psychol..

[44] Westerholm P. (2007). Professional ethics in occupational health. West. Eur. Perspect. Ind. Health.

[45] Black C. (2008). Working for A Healthier Tomorrow.

[46] Cummings K.J., Kreiss K. (2008). Contingent workers and contingent health: Risks of a modern economy. JAMA.

[47] ILO/ISSA/KOSHA (2008). Seoul Declaration on Safety and Health at Work. Safety and Health Summit, XVIII World Congress on Safety and Health at Work: International Labour Organization, International Social Security Association, and Korean Occupational Safety and Health Agency. Ind. Health..

[48] Anttonen H., Räsänen T. (2008). Well-Being at Work: New Innovations and Good Practices.

[49] Punnett L., Warren N., Henning R., Nobrega S., Cherniack M. (2015). CPH-New Research Team. Participatory ergonomics as a model for integrated programs to prevent chronic disease. J. Occup. Environ. Med..

[50] Schulte P.A., Chun H. (2009). Climate change and occupational safety and health: Establishing a preliminary framework. J. Occup. Environ. Hyg..

[51] Virtanen M., Singh-Manoux A., Ferria J.E., Gimeno D., Marmot M.G., Eloveinio M., Joketa M., Vantera J., Kivimäki M. (2009). Long working hours and cognitive function: The whitehall II study. Am. J. Epidemiol..

[52] Johns G. (2010). Presenteeism in the workplace: A review and research agenda. J. Organ Behav..

[53] Bambra C. (2011). Work, worklessness and the political economy of health inequalities. J. Epidemiol. Commun. Health.

[54] Dollard M.F., McTernan W. (2011). Psychosocial safety climates: A multilevel theory of work stress in the health and community service sector. Epidemiol. Psychiatr. Sci..

[55] Asfaw A., Pana-Cryan R., Rosa R. (2012). Paid sick leave and non-fatal occupational injuries. Am. J. Public Health.

[56] Schulte P.A., Pandalai S., Wilson V., Chun H. (2012). Interaction of occupational and personal risk factors in workforce health and safety. Am. J. Public Health..

[57] Kranika-Murray M., Weyman A.K. (2013). Optimising workplace interventions for health and well-being. A commentary on the limitations of the public health approach within the workplace health arena. IJWHM.

[58] Zwetsloot G.I., van Scheppingen A.R., Bos E.H., Dijkman A., Starren A. (2013). The core values that support health safety, and well-being at work. Saf. Health Work..

[59] Bauer G.F., Hämmig O. (2014). Bridging Ocupational, Organizational and Public Health: A Transdisciplinary Approach.

[60] Weil D. (2014). The Fissured Workplace: Why Work Became So Bad for So Many and What Can be Done to Improve It.

[61] Kang S.-K. (2015). New concepts for occupational health development: 3 phrases. Saf. Health Work..

[62] Lentz T.J., Dotson S., Williams P.R.D., Maier A., Gadagbui B., Pandalai S.P., Lamba A., Hearl F., Mamtaz M. (2015). Aggregate exposure and cumulative rich assessment and non-occupation risk factors. JOEH.

[63] Schulte P.A., Guerin R.J., Schill A.L., Bhattacharya A., Cunningham T.R., Pandalai S.P., Eggerth D., Stephenson C.M. (2015). Considerations of incorporating well-being in public policy for workers and workplaces. Am. J. Public Health.

[64] Blustein D.L., Olle C., Connors-Kellgren A., Diamonti A.J. (2016). Decent work: A psychological perspective. Front. Psychol..

[65] DeBord D.G., Carreon T., Lentz T.J., Middendorf P.J., Hoover M.D., Schulte P.A. (2016). The use of the “exposome” in the practice of epidemiology: A primer on omic-technologies. Am. J. Epidemiol..

[66] Harrison J., Dawson L. (2016). Occupational health: Meeting the challenges in the next 20 years. Saf. Health Work..

[67] Dugan A.G., Punnett L. (2017). Dissemination and Implementation research for occupational safety and heats. Occup. Health Sci..

[68] Ganzleben C., Antignac J.-P., Barouki R., Castaño A., Fiddicke U., Klánová J.K., Lebret E., Olea N., Sarigiannis D., Schoeters G.R. (2017). Human biomonitoring as a tool to support chemicals regulation in the European Union. Int. J. Environ. Res. Public Health.

[69] Oeij P.R.A., Rus D., Pot F.D. (2017). Workplace Innovation: Theory, Research and Practice.

[70] Sauter S., Hurrell J.J. (2017). Occupational heath contributions to the development and promise of occupational health psychology. J. Occup. Health Psychol..

[71] Iavicoli S., Valenti A., Gagliardi D., Cantanen J. (2018). Ethics and occupational health in the contemporary world and work. Int. J. Environ. Res. Public Health.

[72] Pfeffer J. (2018). Dying for a Paycheck.

[73] DeJoy D.M., Wilson M.G., Hudson H.L., Nigam J.A.S., Sauter S.L., Chosewood L.C., Schill A.L., Howard J. (2019). Total Worker Health: Evolution of the concept. Total Worker Health^®^.

[74] Hudson H.L., Nigam J.A.S., Sauter S.L., Chosewood L.C., Schiff A.L., Howard J. (2019). Total Worker Health^®^.

[75] ILO (2019). Sustainable Development Goals (SDG): Goal 8. Promote Inclusive and Sustainable Growth, Employment, and Decent Work for All.

[76] Felknor S.A., Streit J.M.K., Chosewood L.C., McDaniel M., Schulte P.A., Delclos G.L. (2020). How will the future of work shape the OSH professional of the future? A workshop summary. Int. J Environ. Res. Public Health.

[77] Newman L.S., Scott J.S., Childness A., Linnan L., Newhall W.J., McLellan D.L., Campo S., Freewynn S., Hammer L.B., Leff M. (2020). Education and training to build capacity in Total Worker Health. JOEM.

[78] Way K. (2020). Psychosocial hazards. The core body of knowledge for generalist OHS professionals.

[79] Guerin R.J., Harden S.M., Rabin B.A., Rohlman D.S., Cunningham T.R., TePoel M.R., Parish M., Glasgow R.E. (2021). Dissemination and Implementation Science Approaches for Occupational Safety and Health Research: Implications for Advancing Total Worker Health. Int. J. Environ. Res. Public Heal..

[80] Lovejoy M., Kelly E., Kubzansky L., Berkman L.F. (2021). Work redesign for the 21st century: Promising strategies for enhancing worker well-being. Am. J. Public Health.

[81] Pratap P., Dickson A., Love M., Zanoni J., Donato C., Flynn M., Schulte P.A. (2021). Public health impacts of underemployment and unemployment in the United States: Exploring perceptions, gaps, and opportunities. Int. J. Environ. Res. Public Health.

[82] Tamers S., Pana-Cryan R., Ruff T., Streit J., Flynn M., Childress A., Chang C.C., Novicki E., Ray T., Fosbroke D. (2022). The NIOSH Future of Work Initiative Research Agenda.

[83] Rogers B., Schill A.L. (2021). Ethics and Total Worker Health^®^: Constructs for ethical decision-making and competencies for professional practice. Int. J. Env. Res. Public Health.

[84] Samuels S.W., Ringen K., Rom W.N., Frank A. (2022). Ethical thinking in occupational and environmental medicine: Commentaries from the Selikoff Fund for Occupational and Environmental Cancer Research. Am. J. Ind. Med..

[85] Fujishiro K., Ahonen E.Q., de Porras D.G.R., Chen I.-C., Benavides F.G. (2021). Sociopolitical values and social institutions: Study in work and health equity through the lens of the political economy. Popul. Health.

[86] Flynn M.A., Check P., Steege A.L., Siven J.M., Syron L.N. (2022). Health equity and a paradigm shift in occupational safety and health. Int. J. Environ. Res. Public Health.

[87] Ilmarinen J., Tuomi K., Klockars M. (1997). Changes in the work ability of active employees over a 11-year period. Scand. J. Work Environ. Health..

[88] Chosewood L.C., Kirby E. (2022). Total Worker Health Program readies NIOSH for the next 50 years. NIOSH Science Blog.

[89] Punnett L., Cavallari J.M., Henning R.A., Nobrega S., Dugan A.G., Cherniak M.G. (2020). Defining ‘integration’ for Total Worker Health: A new proposal. Ann. Work. Expo. Health.

[90] Lovelock K. *Psychosocial Hazards in Work Environments and Effective Approaches for Managing Them*; Worksafe New Zealand. April 2019. https://www.worksafe.govt.nz/research/psychosocial-hazards-in-work-environments-and-effective-approaches-for-managing-them/.

[91] Wild C. (2005). Complementing the genome with an “exposome”: The outstanding challenge of environmental exposure measurement in molecular epidemiology. Cancer Epidemiol. Biomarkers Prev..

[92] NIOSH Exposome and Exposomics.

[93] Gilles L., Govarts E., Martin L.R., Andersson A.-M., Appenzeller B.M.R., Barbone F., Castaño A., Coertjens D., Hond E.D., Dzhedzheia V. (2022). Harmonization of human biomonitoring studies in Europe: Characteristics of HBM4EU-aligned studies participants. Int. J. Environ. Res. Public Health.

[94] Scheepers P.T.J., Duca R.C., Galea K.S., Godderis L., Hardy E., Knudsen L.E., Leese E., Louro H., Mahiout S., Ndaw S. (2021). HBM4EU Occupational biomonitoring study on e-waste—Study, protocol. Int. J. Environv. Res. Public Health.

[95] Pandalai S.P., Schulte P.A., Miller D.B. (2013). Conceptual models of the interrelationship between obesity and the occupational environment. Scand. J. Environ. Health.

[96] World Health Organization (2010). Healthy Workplaces: A Model for Action for Employers, Workers, Policy Makers and Practitioners.

[97] DGUV (2016). New Forms of Work, New Forms of Prevention.

[98] Eeckelaert L., Dhondt S., Oeij P., Pot F., Nicolescu G.I., Webster J., Eisler D. (2012). Review of Workplace Innovation and Its Relation with Occupational Safety and Health.

[99] Karasek R.A., Theorell T. (1990). Healthy Work: Stress, Productivity, and the Reconstruction of Working Life.

[100] Pot F., Dhondt S., Mohr B.J., Van Amelsvoort P. (2016). Workplace innovation. Co-Creating Humane and Innovative Organizations. Evolutions in the Practice of Socio-Technical System Design.

[101] International Labour Organisation Istanbul Declaration on Safety and Health at Work.

[102] Finnish Institute of Occupational Health (2019). Vision Zero 2019.

[103] Geisler E. (1996). An integrated cost-performance model of public sector research evaluation. Scientometrics.

[104] Van Eerd D., Cole D., Keown E., Irvin E., Kramer D., Brennenman-Gibson J., Kazman M., Mahood Q., Slack T., Amick B. (2011). Report on Knowledge Transfer and Exchange Practices: A Systematic Review of the Quality and Types of Instruments Used to Assess KTE Implementation and Impact.

[105] Estabrooks C.A., Derksen L., Winther C., Lavis J.N., Scott S.D., Wallin L., Profetlo M., Grath J. (2008). The intellectual structure and substance of the knowledge utilization field: A longitudinal author co-citation analysis, 1945–2004. Implement. Sci..

[106] Rabin B.A., Brownson R.C., Haire-Joshu D., Kreuter M.W., Weaver N.L. (2008). A glossary for dissemination and implementation research in health. J. Public Health Manag. Pract..

[107] Schulte P.A., Cunningham T.R., Nickels L., Felknor S., Guerin R., Blosser F., Chang C.-C., Check P., Eggerth D., Flynn M. (2017). Translation research in occupational safety and health: A proposed framework. Am. J. Ind. Med..

[108] Schulte P., Okun A., Stephenson C., Colligan M., Ahlers H., Gjessing C., Loos G., Niemeier R., Sweeney M. (2003). Information dissemination and use: Critical components in occupational safety and health. Am. J. Ind. Med..

[109] Cunningham T.R., Tinc P.J., Guerin R.J., Schulte P.A. (2020). Translation research in occupational health and safety settings: Common ground and future directions. J. Saf. Res..

[110] Gewirth A. (1986). Human rights and the workplace. Am. J. Industr. Med..

[111] Yeh M.-J., Liu H.-C. (2018). Comment on Iavicoli et al., Ethics and occupational health in the contemporary world of work. Int. J. Environ. Res. Public Health.

[112] Schulte P.A., Sauter S.L. Work and Well-Being: The Changing Face of Occupational Safety and Health. *NIOSH Science Blog.* 7 June 2021. https://blogs.cdc.gov/niosh-science-blog/2021/06/07/work-and-well-being/.

[113] Stacey N., Ellwood P., Bradbrook S., Reynolds J., Williams H., Lye D. Foresight on New and Emerging Occupational Safety and Health Risks Associated with Digitalization by 2025. https://osha.europa.eu/en/publications/foresightnew-and-emerging-occupational-safety-and-health-risks-associated/view.

[114] Delclos G.L. (2019). Principal Investigator. U13 Cooperative Agreement: Shaping the Future to Ensure Worker Health and Well-Being: Shifting Paradigms for Research, Training and Policy.

[115] Schulte P.A., Vainio H. (2010). Well-being at work: Overview and perspective. Scand. J. Work Environ. Health.

[116] Pratap P. Horizontal expansion: Socioeconomic factors [Panelist]. Proceedings of the Expanded Focus for Occupational Safety and Health (Ex4OSH) International Conference.

[117] Hammer L.B., Brady J.M., Brossoit R.M., Mohr C.D., Bodner T.E., Crain T.L., Brockwood K.J. (2021). Effects of Total Worker Health leadership intervention on employee well-being and functional impairment. J. Occup. Health. Psychol..

[118] Cengiz D., Dube A., Linder A., Zipperer B. (2019). The effect of minimum wages on low-wage jobs. Q. J. Econ..

[119] Chari R., Sauter S.L., Petrun Sayers E.L., Huang W., Fisher G.G., Chang C.C. (2022). Development of the National Institute for Occupational Safety and Health Worker Well-being Questionnaire. J. Occup. Environ. Med..

[120] MacDonald L.A., Härenstam A., Warren N.A., Punnett L. (2008). Incorporating work organization into occupational health research: An invitation for dialogue. Occup. Environ. Med..

[121] Miranda H., Gore R.J., Boyer J., Nobreya S., Punnett L. (2015). Health behaviors and overweight in nursing home employees: Contribution of workplace stressor and implications for worksite health promotion. Sci. World J..

[122] Peters S.E., Denerlein J.T., Wagner G.R., Sorensen G. (2022). Work and worker health in the post pandemic world: Public health perspectives. Lancet..

[123] Chandola T., Britton A., Brunner E., Hemingway H., Malik M., Kumari M., Badrick E., Kivimaki M., Marmot M. (2008). Work stress and coronary heart disease: What are the mechanisms?. Eur. Heart, J..

[124] Ishizaki M., Nakagawa H., Morikawa Y., Honda R., Yamada Y., Kawakami N. (2008). Japan work stress and health cohort study group. Influences of job strain on changes in bay mass index and waist circumference—6 year longitudinal study. Scan J. Work. Environ. Health.

[125] Nobrega S., Kernan L., Plaku-Alakbarora B., Robertson M., Warren N., Henning R. (2017). CPH-NEW Research Team. Field tests of a participatory ergonomics toolkit for Total Worker Health. Appl. Ergon..

[126] Cherniack M., Berger S., Namazi S., Henning P., Punnett L. (2019). A particaptory action research approach to mental health interventions among corrections officers: Standardizing priorities and maintaining design autonomy. Occup Health Sci.

[127] El Ghazari M., Jaegers L.A., Monteiro C.E., Grubb P.L., Cherniac K. (2020). Progress in corrections worker health: The national corrections collaborative utilizing a Total Worker Health® strategy. Occup. Environ. Med..

[128] Punnett L. Expanding our focus: From what to what. Proceedings of the Expanded Focus for Occupational Safety and Health (Ex4OSH) International Conference.

[129] Greenwood K., Anas J. (2021). It’s a new era for mental health at work. *Harvard Business Review*. https://hbr.org/2021/10/its-a-new-era-for-mental-health-at-work.

[130] Bakker A.B., Cost P.L. (2014). Chronic job burnout and daily functioning: A theoretical analysis. Burn. Res..

[131] Schulte P.A., Pana-Cryan R., Schnorr T., Schill A.L., Guerin R., Felknor S., Wagner G.R. (2017). An approach to assess the burden of work-related injury, disease, and distress. Am. J. Public. Health.

[132] Engel G.L. (1977). The need for a new medical model: A challenge for biomedicine. Science.

[133] Jenny G.J., Bauer G.F., Vinje H.F., Vogt K., Torp S., Lindstrom B., Espnes G.A., Torp S., Mittlemarch M.B., Sagy S., Mittelmark M.B., Sagy S., Eriksson M., Bauer G.F., Pelikan J.M., Lindström B., Espnes G.A. (2017). The Application of Salutogenesis to Work. The Handbook of Salutogenesis.

[134] Bauer G.F., Roy M., Bakibinga P., Contu P., Downe S., Eriksson M., Espnes G.A., Jensen B.B., Juvinya Canal D., Lindstrom B. (2020). Future directions for the concept of salutogenesis: A position article. Health Promo. Intl..

[135] Walsh M. Day 2 opening address. Proceedings of the Expanded Focus for Occupational Safety and Health (Ex4OSH) International Conference.

[136] Goldin I., Muggah R. (2020). COVID-19 is Increasing Multiple Kinds of Inequality. Here’s What We Can Do About It..

[137] Baron S.L., Beard S., Davis L.K., Delp L., Forst L., Kidd-Taylor A., Liebman A.K., Linnan L., Punnett L., Welch L.S. (2014). Promoting integrated approaches to reducing health inequities among low-income workers: Applying a social ecological framework. Am. J. Ind. Med..

[138] Do D.P., Frank R.U.S. (2021). frontline workers and COVID-19 inequities. Prev. Med..

[139] Piketty T. (2014). Capital in the Twenty-First Century.

[140] Leonhardt D. Our broken economy, in one simple chat. *New York Times*, 7 August 2017. https://www.nytimes.com/interactive/2017/08/07/opinion/leonhardt-income-inequality.html.

[141] Chancel L., Piketty T., Saez E., Zucman G. (2021). World Inequality Report 2022.

[142] Picketty T., Saez E., Zucman G. (2016). Distributional National Accounts; Methods and Estimates for the United States.

[143] Bryson A., Forth J., Stokes L. (2018). The performance pay premium and wage dispersion in Britain. The Manchester School. City Res. Online.

[144] Solar O., Irwin A. (2010). A Conceptual Framework for Action on the Social Determinants of Health.

[145] Flynn M. Work, mental health, and well-being: A biopsychosocial approach to health. Proceedings of the Expanded Focus for Occupational Safety and Health (Ex4OSH) International Conference.

[146] Pateman C. (1970). Participation and Democratic Theory.

[147] Eldor L., Harpaz I., Westman M. (2020). The Work/Nonwork Spillover: The Enrichment Role of Work Engagement. J Lead. Org Stud..

[148] Meadows D.H. (2008). Thinking in Systems.

[149] Carey G., Malbon E., Carey N., Joyce A., Crammond B., Carey A. (2015). Systems science and systems thinking for public health: A systematic review of the field. BMJ Open.

[150] Nicolson C.R., Starfield A.m., Kofinas G.P., Kruse J.A. (2002). 2002. Ten heuristics for interdisciplinary modeling projects. Ecosystems.

[151] Streit J.M.K., Felknor S.A., Edwards N.T., Howard J. (2021). Leveraging strategic foresight to advance worker safety, health, and well-being. Int. J. Environ. Res. Public Health.

[152] Streit J., Felknor S.A., Edwards N.T., Howard J. Advancing worker safety, health, and well-being with strategic foresight. Proceedings of the Expanded Focus for Occupational Safety and Health (Ex4OSH) International Conference.

[153] Shaw W.S., Roelofs C., Punnett L. (2020). Work environment factors and presentation of opioid-related deaths. Am. J. Public Health..

[154] Hawkins D., Davis L., Punnett L., Kriebel D. (2020). Disparities in the deaths of despair by occupation, Massachusetts, 2005 to 2015. J. Occup. Environ. Med..

[155] Henning R., Wallen N., Robertson M., Faghri P., Chorniack M., The CPH-New Research Team (2009). Workplace health protection and promotion through participatory ergonomics: An integrated approach. Public Health Rep..

[156] Haines M., Wilson J.R., Vink P., Koningsueld E. (2002). Validating a framework for participatory ergonomics (the PEF). Ergonomics.

[157] Green L., George A.M., Marek D., Frankish J.C., Herbert C.P., Bowle W., Minkler M., Wellerstein N. (2003). Guidelines to participatory research in health promotion. Community-Based Participatory Research for Health.

[158] Loewenson R., Laurell A.C., Hogstedt C. (1995). Participatory approaches in occupational health research: A review. Med. Lav..

[159] Mergler D. (1987). Worker participation in occupational health research: Theory and practice. Int. J. Health Serv..

[160] Lacouture A., Breton E., Guichara A., Riddle S. (2015). The concept of mechanism from a realist approach: A scoping review to facilitate its operationalization in public health program evaluation. Implement. Sci..

[161] Nurjono M., Shrestha P., Lee A., Lim X.Y., Shiraz F., Tan S., Wong S.H., Foo K.M., Wee T., Toh S.-A. (2018). Realist evaluation of a complex integrated care programme: Protocol for a mixed methods study. BMJ Open.

[162] Eurofound and Cedefop (2020). European Company Survey 2019: Workplace Practices Unlocking Employee Potential.

[163] Eurofound, Cedefop (2021). Innovation in EU Companies: Do Workplace Practices Matter? European Company Survey 2019 series.

[164] Schnall P., Dobson M., Rosskam E. (2009). Unhealthy Work: Causes, Consequences, Cures.

[165] Rodrik D., Sabel C.F. (2020). Building a Good Jobs Economy.

[166] Evanoff B.A., Bohrf K., Wolf L.D. (1999). Effects of a participatory ergonomics team among hospital orderlies. Am. J. Ind. Med..

[167] Sinclair R.C., Cunningham T.R., Schulte P.A. (2015). A model for occupational safety and health intervention diffusion to small businesses. Am. J. Ind. Med..

[168] Jaegers L., Dale A.M., Weaver N., Buckholz B., Welch L., Evanoff B. (2014). Development of a program logic model and evaluation plan for a participatory ergonomics intenention in construction. Am. J. Ind. Med..

[169] Roelofs C., Sugerman-Brozan J., Kurowski A., Russell L., Punnett L. (2021). Promoting opioid awareness through a union-based peer training model. New Solut..

[170] Warr P.B. (1990). Decision latitude, job demands, and employee well-being. Work. Stress.

[171] Weziak-Baialowolska D., Bialowolski P., Sacco P.L., Wanderweele T.J., McNeely E. (2020). Well-being in life and well-being at work: Which comes first? Evidence from a longitudinal study. Front. Public Health.

[172] Peters S.E., Sorensen G., Katz J.N., Gunderson D.A., Wagner G.R. (2021). Thriving from work: Conceptualization and measurement. Int. J. Environ. Res. Public Health.

[173] Chirico F., Heponiemi T., Parlou M., Zaffing S., Magnavita N. (2019). Psychosocial risk prevention in a global health perspective. A descriptive analysis. Int. J. Environ. Res. Public Health..

[174] Schnall P.L., Landsbergis P.A., Baker D. (1994). Job strain and cardiovascular disease. Ann. Rev. Public Health.

[175] Niedhammer I., Bertrais S., Witt K. (2018). Psychosocial work exposures and health outcomes: A meta-review of 72 literature reviews with meta-analysis. Scand. J. Work. Environ. Health.

[176] Thiede I., Thiede M. (2015). Quantifying the costs and benefits of occupational health and safety interventions at a Bangladesh shipbuilding company. Int. J. Occup. Environ. Med..

[177] Jain A., Leka S., Zwetsloot G. (2021). Managing Health Safety and Well-Being. Ethics, Responsibility and Sustainability.

[178] Oakman J., Kinsman N., Stucheg R., Graham M., Woale V. (2020). A rapid review of mental and physical health effects of working at home: How do we optimise health?. BMC Public Health.

[179] Strecher V. (2016). Life on Purpose: How Living for What Matters Most Changes Everything.

[180] Cocker F., Sanderson R., La Montagne A.D. (2017). Estimating the economic benefits of eliminating job strain as a risk factor for depression. J. Occup. Environ. Med..

[181] Bremen J.M., Levanat A.D., Stretcher V., Zimmerman E. (2020). Building Dignity and Purpose to Create Greater Performance During the Pandemic. https://www.wtwco.com/en-US/Insights/2020/10/building-dignity-and-purpose-to-create-greater-performance-and-wellbeing.

[182] Strecher V. Employee Mental Health, Work Engagement, and Retention in a New Era: A New Model, New Interventions. Proceedings of the Expanded Focus for Occupational Safety and Health (Ex4OSH) International Conference.

[183] Stahl A. What the Future of Work Means for Our Mental Health. *Forbes*
**2020**. https://www.forbes.com/sites/ashleystahl/2020/10/09/what-the-future-of-work-means-for-our-mental-health/.

[184] Schaufeli W.B., Peeters M.C. (2000). Job stress and burnout among correctional officers. A literature review. Int. J. Stress Mgmt..

[185] Parker S.K., Morgeson F.P., Johns G. (2017). One hundred years of work design research: Looking back and looking forward. J Appl. Psychol.

[186] Rössler W. (2012). Stress, burnout, and job dissatisfaction in mental health workers. Eur. Arch. Psychiatry Clin. Neuro. Sci..

[187] Hola B., Nowobilski T. (2019). Analysis of the influence of socio-economic factors on occupational safety in the construction industry. Sustainability.

[188] CSA Group (2013). Stand-Alone Guidance: Psychological Health in the Workplace.

[189] International Standards Organization (2021). ISO 45003: Occupational Health and Safety Management—Psychological Health and Safety at Work—Guidelines for Managing Psychosocial Risks.

[190] Dollard M.F., Bailey T. (2021). Building a psychosocial safety climate in turbulent times: The case of COVID-19. J. Appl. Psych..

[191] Dollard M.F., Tuckey M.R., Dormann C. (2012). Psychological safety climate moderates the job demand resource interaction in predicting work group distress. Accid. Anal. Prev..

[192] Miller M. (1989). Social, economic, and political forces affecting the future of occupational health nursing. AAOHN J..

[193] Brynjolfsson E., McAfee A. (2014). The Second Machine Age: Work, Proper Prosperity in a Time of Brilliant Technologies.

[194] Cain C. Future of work: Partnerships, perspectives, policy, and practice. Proceedings of the Expanded Focus for Occupational Safety and Health (Ex4OSH) International Conference.

[195] Lauer M. (2014). The Future of Work Requires a Return to Apprenticeships.

[196] Osterman P. (2019). Employment and Training for Mature Adults: The Current System and Moving Forward.

[197] Lagerlöf E. (2000). Research dissemination: Proceedings of a workshop in Brussels 24th November 1998. Arb. Och Hälsa.

[198] Choi S.L., Heo W., Cho S.H., Lee P. (2020). The links between job insecurity, financial well-being and financial stress: A moderated mediation model. Int. J. Consum. Study.

[199] Main C.J., Shaw W.S., Nicholas M.K., Linton S.J. (2022). System-level efforts to address pain-related workplace challenges. Pain.

[200] Siqueira C.E., Gaydos M., Monforton C., Slatin C., Borkowski L., Dooley P., Liebman A., Rosenberg E., Shor G., Keifer M. (2014). Effects of social, economic, and labor policies on occupational health disparities. Am. J. Ind. Med..

[201] Piha K., Laaksonen M., Martikainen P., Rahkonen O., Lahelma E. (2012). Socio-economic and occupational determinants of work injury absence. Eur. J. Public Health.

[202] Bushnell T. Economic factors in safety, health and well-being and the roles of public policy. Proceedings of the Expanded Focus for Occupational Safety and Health (Ex4OSH) International Conference.

[203] Pot F., Abrahamsson K., Ennals R. (2022). Monotonous and Repetitive Work—Some People are more unequal Than Others.

[204] Barth A., Winker R., Ponocny-Seliger E., Sögner L. (2007). Economic growth and the incidence of occupational injuries in Austria. Wien. Klin. Wochenschr..

[205] Silver S.R., Li J., Quay B. (2022). Employment status, unemployment duration, and health-related metrics among US adults of prime working age: Behavioral Risk Factor Surveillance System, 2018–2019. Am. J. Ind. Med..

[206] Waring A. (2019). The five pilars of occupational safety & health in a context of authoritarian soci-politcal climate. Saf. Sci..

[207] The Lancet (2009). A commission on climate change. Lancet.

[208] Applebaum K.M., Graham J., Gray G.M., LaPumu P., McCormick S.A., Northcross A., Perry M.J. (2016). An overview of occupational risks from climate change. Curr. Environ. Health Rep..

[209] Schulte P.A., Battacharya A., Butter C.R., Chun H.H., Jacklitsch B., Jacobs T., Kiefer M., Lincoln J., Pendergrass S., Shire J. (2016). Advancing the framework for considering the effects of climate change on worker safety and health. J. Occup. Environ. Hyg..

[210] Kjellsrom T., Maitre N., Saget C., Otto M., Karimova T. (2019). Working on a Warmer Planet: The Impact of Heat Stress on Labour Productivity and Decent Work.

[211] Roelofs C., Wegman D. (2014). Workers: The climate canaries. Am. J. Public Health.

[212] Charlson F., Ali S., Bermarhnia T., Pearl M., Massozza A., Augustinavicius J., Scott J. (2021). Climate change and mental health: A scoping review. Int. J. Environ. Res. Public Health.

[213] Azzi M. Managing work-related psychosocial risks during the pandemic and beyond. Proceedings of the Expanded Focus for Occupational Safety and Health (Ex4OSH) International Conference.

[214] International Labour Organisation (2020). Managing Work-Related Psychosocial Risks During the COVID-19 Pandemic.

[215] de Jong T., Wiezer N., De Weerd M., Nielsen K., Mattila-Holappa P., Mockallo Z. (2016). The impact of restructuring on employee well-being: A systematic review of longitudinal studies. Work. Stress.

[216] Schaufeli W.B., Bakker A.B., Van Rhenen W. (2007). How changes in job demands and resources predict burnout, work engagement, and sickness absenteeism. J. Organ. Behav..

[217] Van der Weele T.J., Fulks J., Plake J.F., Lee M.T. (2021). National well-being measures before and during the COVID-19 pandemic in online samples. J. Gen. Intern. Med..

[218] Dennerlein J. Improving COVID-19 policies and practices using total worker ealth approaches for essential workplaces: A case study in the energy supply sector. Proceedings of the Expanded Focus for Occupational Safety and Health (Ex4OSH) International Conference.

[219] Leff M. Assessing the impact of COVID-19 on small and medium sized businesses: The Carolina PROSPER worksite impact survey. Proceedings of the Expanded Focus for Occupational Safety and Health (Ex4OSH) International Conference.

[220] Hines A., Bishop P. (2015). Thinking about the Future.

[221] Bishop P., Hines A. (2012). Teaching about the Future.

[222] NIOSH Strategic Foresight at NIOSH.

[223] Lax M.B. (2016). The perils of integrating wellness and safety and health and possibility of a worker-oriented alternative. New Solut..

