# Supplementary material for: Expanding the Focus of Occupational Safety and Health: Lessons from a Series of Linked Scientific Meetings
Source: Int J Environ Res Public Health. 2022 Nov 21;19(22):15381. doi: 10.3390/ijerph192215381 (PMC9690540; doi:10.3390/ijerph192215381)
Supplement: Supplementary file 1 [file ijerph-19-15381-s001.zip › ijerph-1984796-supplementary.pdf]

Supplementary Materials *

Expanding the Focus of Occupational Safety and Health: Lessons from a Series of Linked Scientific Meetings

Paul A. Schulte, George L. Delclos, Sarah A. Felknor, Jessica M.K. Streit, Michelle McDaniel, L. Casey Chosewood, Lee S. Newman, Faiyaz A. Bhojani, Rene Pana-Cryan and Naomi G. Swanson

* All references cited can be found in the reference list of the main article.

1. Historical Influences on an expanded focus for occupational safety and health

Calls for the OSH field to evolve are not new. Rantanen, in 1999 [35], explored new challenges for occupational health research and observed that "macro and micro-scale trends will substantially change the conditions under which research in occupational health will be carried out." Rantanen [35] noted that "the dynamic changes in the structure of work life make collection of data on exposures, hazards, the health of working people, and the impact of control measures more difficult than in the past."

Prior to Rantenen's observations, two other efforts set the stage for thinking about the need to consider a broader focus for OSH. First, Ilmarinen and colleagues [87] investigated how changing age structure and workability required a multidisciplinary approach to search for new concepts of work and retirement. Workability is defined as the balance between human resources and the demands of work, which can be quantified in the Workability Index (WAI) [26,87]. Coggon [9] concluded that "…much of today's occupational illness is not a simple function of excessive exposure to noxious agents or activities," and a new approach was needed. This approach calls for research that addresses the understanding "…that much of illness and disability that is attributed to injurious occupational exposures does not arise from underlying disease with detectable organic pathology but rather is a psychologically mediated response to an external trigger that is conditional by a combination of individual characteristics and cultural circumstances" [9].

More broadly, the Finnish Institute of Occupational Health developed a new integrated concept of well-being at work based on dialogue between different groups and social partners (Occupational Safety and Health [OSH], Occupational Health Services [OHS], Human Resource Development [HRD], and Business Management) in the workplace. The concept involved a shift from a multidisciplinary to an interdisciplinary, coherent way of action [48]. "Well-being at work is an approach of comprehensive actions concerning the promotion of better health, safety and well-being of the workforce, simultaneously with the promotion of productivity and success of the company or enterprise," Anttonen and Räsänen noted [48]. In 2015, Schulte at al. [63] called for a strategy for conducting research to fill gaps in the evidence base for what works and does not work in achieving or maintaining well-being and acknowledged that "these efforts will hinge on clarifying the constituent factors that contribute to well-being as well as identifying promising interventions to address or enhance well-being."

1.1. Prevention and Promotion

A critical report inspiring thinking about the evolution of OSH was "Working for a Healthier Tomorrow," by Black, in 2008 [45]. The report concluded that "a shift in attitudes is necessary to ensure that employers and employees recognize not only the importance of preventing ill-health but also the key role the workplace can play in promoting health and well-being at work is most needed." The report set out a new approach to support the health and well-being of all working age people, which would depend on "…having a workforce of health professionals who are equipped to meet its current and

future needs. For this they need the right skills, evidence base and organizational structures [45].”

In parallel with the report by Black [45] and building on work in the 1980s, the Total Worker Health® (TWH)concept was fostered in the United States [73]. TWH was built on the principle that simultaneously addressing OSH and Workplace Health Promotion (WHP) would create a “synergy of protection.” The ultimate goal of TWH involves moving the OSH field forward by defining, enhancing, and operationalizing the concept of “well-being” in the context of work, as well as envisioning work “that enhances all aspects of daily life, thinking beyond work as a means of survival, to envision work as a way of providing meaning, fulfillment, and improved health and well-being” [88]. TWH training overlaps OSH training in some ways but may be seen as different [77]. Nonetheless, an expanded focus for the OSH field would include a TWH approach.

1.2. Work Relatedness

Promoting thinking that goes beyond established occupational disease is not new. In 1984, WHO initiated a program on work-related diseases, defined as multifactorial diseases in which “the work environment plays a pivotal role in causation” [22]. Work-relatedness of multifactored diseases, El Batawi [22] noted, “comes from the fact that human life at home, at work, and during leisure time is a continuity that does not recognize the boundaries that separate the work environment from the general environment. It also stems from the fact that heredity and lifestyle factors influencing human health do not stop at the gates of the workplace.” WHO called for training programs that introduce elements of work-related diseases in occupational health curricula. Work-related diseases and injury may be seen to exist on a continuum of attributable risks, with complete work-relatedness on one extreme and complete non-work-relatedness on the other extreme; the midpoint of the continuum can be envisioned as an attributable risk of approximately 50%.

The TWH program acknowledged the boundary between the porosity between work and non-work factors; it called for “… a holistic understanding of the factors that contribute to worker well-being … Risk factors in the workplace can contribute to health problems previously considered unrelated to work” [89].

1.3. Attention to Psychosocial Hazards

Historically the OSH field’s focus was on physical, chemical, biological, and ergonomic hazards. However, there is a rich history of addressing the way work was organized and the psychological impact of work on workers. The 1979 study by Karasek [21] on job demands and controls is a foundational contribution to understanding psychosocial stressors. Other models and insights followed [87,88]. In 1991, Sauter et al. [25] presented a strategy to prevent work-related psychological disorders. Cox and colleagues (2000) [8] presented an in-depth inventory of psychosocial hazards related to stress. The growth of regulations and practices to assess and control psychosocial hazards has increased [10] and been linked to the concept of decent work [64]. Despite this history, there have been few evaluations of interventions to control psychosocial hazards or utilize them [90].

1.4. Exposome, Cumulative Risk Assessment, and Human Biomonitoring

Another expansion in OSH is in the areas of the exposome and cumulative risk assessment. Wild [91] described the exposome as the totality of exposures individuals experience from conception to death and its impact on chronic diseases. “Exposures can include toxicants in the general environment and in workplaces, diet, lifestyle choices and even socioeconomic status,” noted DeBord et al. [65]. “The concept of an exposome and specifically, the occupational exposome, represents a holistic approach for investigating worker safety and health” [65,92].

One approach to advancing the exposome and cumulative risk assessment is the collection of data on chemical exposure to workers and the general population. The concept

of 'human biomonitoring' has a long history in occupational health but large national surveys such as the U.S. National Health and Nutrition Examination Survey (NHANES, https://www.cdc.gov/visionhealth/vehss/data/national-surveys/national-health-and-nutrition-examination-survey.html), the Korean National Survey for Environmental Pollutants in the Human Body (KorSEP, http://exposome-explorer.iarc.fr/cohorts/88), and Human Biomonitoring Initiative (HBM4EU, https://www.hbm4eu.eu/) are relatively recent [93]. These surveys are necessary to provide perspective on worker exposures [94]. The HBM4EU is a novel approach to collaborating between various EU agencies and countries and illustrates how research funding can build links between research and policy [93].

While there are many challenges to using exposome data, it is likely to enhance exposure science and broaden the focus of investigators. In 2003, in a parallel effort, the U.S. EPA [37] defined cumulative risk as the combination of risks posed by aggregate exposure to multiple agents or stressors in which aggregate exposure is by all routes and pathways and from all sources of a given agent and stressor. According to Lentz et al. [62], "This approach extends beyond chemicals to include psychosocial, physical and other factors, and provides population-based assessments rather than source-based assessments." The cumulative risk assessment concept is in its infancy and will require identification and development of more assessment metrics, aggregation methods, and approaches based on multiple sources, pathways, and routes [62].

The multifactorial nature of most diseases, injuries, and other health conditions, while generally recognized in OSH, has not been utilized in OSH research, preventive interventions, or policy. In part this was because nonwork factors generally are treated as confounders, notably in research that usually has involved a single risk factor. This approach has led to a lack of comprehensive research on the combined role of work and nonwork risk factors. To address this dearth of studies and potential resulting knowledge, Schulte et al. [56] developed 32 examples of four heuristic combinatorial models, of occupational hazards, and personal risk factors (genetics, age, gender, chronic diseases, obesity, smoking, alcohol use, and prescription drug use). A more expanded assessment was conducted for obesity and occupational hazards [95] and for opioids and benzodiazepines [96]. These efforts serve as part of the scientific foundation for comprehensive approaches such as Total Worker Health [74].

1.5. International Efforts

Internationally, there has been a call for comprehensive and systems-level approaches to better address risks to safety, health, and well-being of workers [74]. This is illustrated by various efforts. As noted, the work of Black (2008) in the United Kingdom was seminal [45]. Other critically important efforts are the WHO (2010) Healthy Workplaces: A Model for Action [96], the Deutsche-Gesetliche Unfallversicherung (DGUV) position article in 2016 [97], and the 2012 review by the European Agency for Safety and Health at Work of workplace innovation and occupational health and safety [98].

The WHO model [96] is a landmark document based on a systematic review of healthy workplace programs in the global literature. It builds on the broad WHO definition of health and reflects on "how understanding of occupational health has evolved from an exclusive focus on the physical work environment to inclusion of psychosocial and personal health practice factors." The report highlights the importance of integrating OSH wellness and human resource efforts in an enterprise and realizing that a healthy workplace is influenced by the physical work environment, the psychosocial work environment, personal health resources, and the enterprise community involvement [96]. The DGUV report [97] cautions that a risk assessment that only considers individual aspects is ineffective in the digital world of work. A holistic risk assessment, on the other hand, considers all relevant risk factors and their interactions. The report also called for a well-established culture of prevention in a company.

Another example of the expanded focus of OSH is the EU-OSHA review of workplace innovation [98] and its relationship with occupational safety and health. Workplace innovation is defined as "…strategically induced participatory adopted changes in an

organization’s practices of managing, organizing, and deploying human and non-human resources that lead to simultaneously improved organizational performance and improved quality of working life” [69]. Workplace innovation is considered as renewal at the organization level (work organization, HRM and labor relations) and addresses the quality of working life (stress reduction, competence development, organizational participation) and organizational performance (productivity, innovative capacity) simultaneously. Much of the theoretical backgrounds for the workplace innovation approach are the “job-demand-control model”[99] and the sociotechnical systems design [100] “Active jobs” (high demands/high control) not only reduce the risk of work-related stress but also inhibit learning (problem solving, competence development, innovation). While it is complicated to bring workplace innovation concepts and OSH together, they represent cross-cutting functions that are intertwined, and understanding their interrelationship expands the focus of OSH.

The formalization of broader concepts of OSH came about in the 2008 Seoul Declaration [47], which recognized safety and health at work as a fundamental human right and was further expanded in the Istanbul Declaration [101], which called for building a global prevention culture for a healthy and safe future. These holistic concepts were crystalized into a Vision Zero effort to eliminate all accidents and injuries in workers, and in the first Vision Zero Summit 2019 [102] as “a new workplace prevention strategy and a new holistic mind set which is based on the belief that all work injuries can be prevented if the health, safety and well-being of people comes first.” The Vision Zero concept had various historic roots but was launched at the XXI Congress of Safety and Health at Work in 2017.

1.6. Dissemination and Implementation Science

The OSH field has generally emphasized etiologic research, and to some extent intervention research, but rarely has there been emphasis on studies of how to get new knowledge and interventions into practice and assess their impact. Such approaches require moving beyond ‘hazard and exposure’ thinking and often involve considering the socio-economic and political contexts [19,27,79,98].

As part of the Swedish Work Life 2000 program, Lagerlöf concluded that “one of the greatest problems in the occupational safety and health community is the lack of appropriate emphasis on the research involved in dissemination, adaptation and utilization of information [1981]. Around this time, investigators at the Institute for Work and Health in Toronto were examining how organizations could more effectively transfer knowledge to decision-makers (managers and authorities). They assessed the process of transferring research information and knowledge and developed a framework for knowledge transfer and exchange [38,104,105]. These effects paralleled a growing consideration of research on research transfer/translation in the health field, in general, and particularly involving cancer [106]. In the occupational safety and health field in 2017, Dugan and Punnett and Schulte et al. called for the use of dissemination and implementation (D&I) research [67,107]. This involved applying scientific methods to study the transfer and utilization of information. Prior to this call, others have identified the need for such research [28,38,103,108,109]. Translation and D&I research were extensively addressed in the second preparatory workshop in 2000 [19]. More recently, in 2021 Guerin et al. synthesized the existing D&I scientific literature to provide OSH and Total Worker Health investigators and practitioners with an overview of the field and approaches that could be used to move worker protection and health promotion into widespread and sustained practice [79].

1.7. Ethical Foundations of OSH

Development of the OSH field has been predicated on the growing realization of the rights of workers and the struggles to maintain them [36,71,83,84,110,111]. The issue of worker rights is often clouded by dual loyalties where OSH professionals have simultaneous obligations, explicit or implicit, to a third party, usually a private employer or by cost-benefit analytical thinking [36]. Understanding the ethical basis for OSH has been

and will continue to be a major dilemma for the field. A significant foundational examination of this topic is the edited volume by Samuels [23] in 1986, "The environment of the workplace and human values," which concludes that in regard to actions to protect workers at risk, "a fundamentally necessary difference exists between ethics and science…" Due to uncertainty in science, ethics must predominate. When there is uncertainty about hazards the ethical approach is to lean toward protecting the workers. More recently, Iavicoli et al. [71] called for broader attention to ethical issues in OSH and that OSH professionals need to be equipped with the analytical skills to deal with the ethical challenges of everyday practice, research, and policy making [111]. They identified an "integrated approach" to assess the importance of individual professional and institutional ethics and identified "drivers and barriers for correct professional ethics." The tensions between future work and ethical pressures are quite likely and the OSH field will need to further evolve to address it. The future of work will be characterized by technological innovation; lack of control, privacy, and individual autonomy; global pressures; decentralized work; economic disparities; climate-related hazards, and migration of workers [3,36,71,112,113] all of which will present ethical issues to the OSH practitioner.

Table S1. Selected history of contributions to an expanding focus for occupational safety and health[1.]

Year	Author(s)	Topic	Ref ID
1977	Ferguson	Psychology and occupational health	20
1979	Karasek	The role of job demands and control in stress	21
1984	el Batawi	Work-relatedness of multifactorial diseases	22
1986	Samuels	The workplace and human values	23
1987	Antonovsky	Salutogenesis: health promoting factors at work	24
1990	Sauter et al.	Strategy to prevent work-related psychological disorders	25
1991	Ilmarinen et al.	Work ability	26
1992	Amick et al.	Work stressors over the life course	27
1992	Israel et al.	Need for action research	28
1995	Heaney et al.	Enhance worker mental health	29
1995	Sorensen et al.	Integration of health protection and health promotion	30
1996	Quick	Editorial in first issue of *Occupational Health Psychology*	31
1996	Siegrist	High effort/low rewards model	32
1999	Adkins et al.	Occupational health psychology-family interface	33
1999	Dembe	Social inequalities in OSH	34
1999	Rantanen	Conditions under which OSH research will change	35
2000	Cox et al.	Psychosocial hazards	8
2002	London and Kisting	Deep examination of ethics in OSH	36
2003	U.S. EPA	Cumulative risk assessment	37
2003	Lavis et al.	How research can be better transferred	38
2003	Quinn	Connecting OSH with public health	39
2004	Putnam et al.	Managing workplace depression-related: a new opportunity	40
2005	Coggon	Occupational medicine at a turning point	9
2006	Caruso et al.	Long work hours and OSH	41
2006	Schulte	Time for a holistic approach	42
2007	Bakker and Demerouti	Job demands-resources model	43
2007	Westerholm	Ethics in OSH	44
2008	Black	Approach to address, health and well-being of working people	45
2008	Cummings and Kreiss	Contingent worker	46
2008	ILO/ISSA/KOSHA	Seoul declaration: safe work is a human right	47
2009	Anttonen and Rasanen	Work and well-being	48
2009	Punnett et al.	Macroergonomics	49

2009	Schulte and Chun	Framework for climate-related hazards	50
2009	Virtanen et al.	Long work hours/cognitive function	51
2010	Johns	Presenteeism in the workplace	52
2010	Leka and Jain	Addressing psychosocial hazards	10
2011	Bambra	Work: a determinant of health inequalities	53
2011	Dollard and McTernan	Psychosocial safety climate	54
2012	Asfaw et al.	Paid sick leave and occupational injuries	55
2012	Schulte et al.	Interaction: occupational and personal risk factors	56
2013	Kranika-Murray and Weyman	Consideration of a public health approach	57
2013	Zwetsloot et al.	Core values in OSH	58
2014	Bauer and Hämmig	Transdisciplinary approach	59
2014	Weil	Impact of work reorganization on health	60
2015	Kang	New concepts for occupational health development	61
2015	Lentz et al.	Cumulative risk assessment for workers	62
2015	Schulte et al.	Call to operationalize well-being for workers	63
2016	Bluestein et al.	Psychological perspective on decent work	64
2016	DeBord et al.	Exposome	65
2016	Harrison and Dawson	Call for a new paradigm	66
2017	Dugan and Punnett	Call for implementation science in OSH	67
2017	Ganzeleben et al.	Human Biomonitoring	68
2017	Howard	Nonstandard work arrangements	7
2017	Oeij et al.	Workplace innovation theory, research and practice	69
2017	Peckham et al.	Creating the future for OSH	2
2017	Sauter and Hurrell	Occupational health psychology	70
2018	Iavicoli et al.	Ethics in the contemporary world of work	71
2018	Pfeffer	Human sustainability	72
2019	Dejoy and Wilson	Total worker health: evolution of the concept	73
2019	Hudson et al.	State-of-the-art of Total Worker Health	74
2019	ILO	Looking to the future of OSH	75
2019	Schulte et al.	Expand focus of OSH	18
2020	Felknor et al.	Shaping the OSH profession of the future	76
2020	Newman et al.	Training for TWH	77
2020	Schulte et al.	Potential scenarios: the future of work	3
2020	Way	Psychosocial hazards in OSH	78
2021	Guerin et al.	Dissemination and implementation science in OSH	79
2021	Lovejoy et al.	Work redesign for 21st century	80
2021	Felknor et al.	OSH Research and practice in the future	19
2021	Pratap et al.	Unemployment and underemployment	81
2021	Tamers et al.	Research agenda: future of work	82
2021	Rogers and Schill	Ethics and Total Worker Health	83
2022	Samuels et al.	Historic development of ethical thinking in OSH	84
2021	Fujishiro et al.	Social influences on work and health	85
2022	Flynn	Health equity and a paradigm shift	86

[1] Selected history based on authors' assessment of novelty and importance of scientific literature. The history of an expanded focus for OSH involves conceptual, psychological, technical, and ethical expansions and addresses only the modern era (after 1970). It is realized that earlier in the history of OSH there were expansive ideas not the least of which is from Ramazzini in De Morbis Artficum: "I admit that the work which I am about to publish is imperfect, or rather is merely intended to incite others to extend a helping hand until we can obtain a really complete a thorough treatise…" (Preface, 1713 edition).

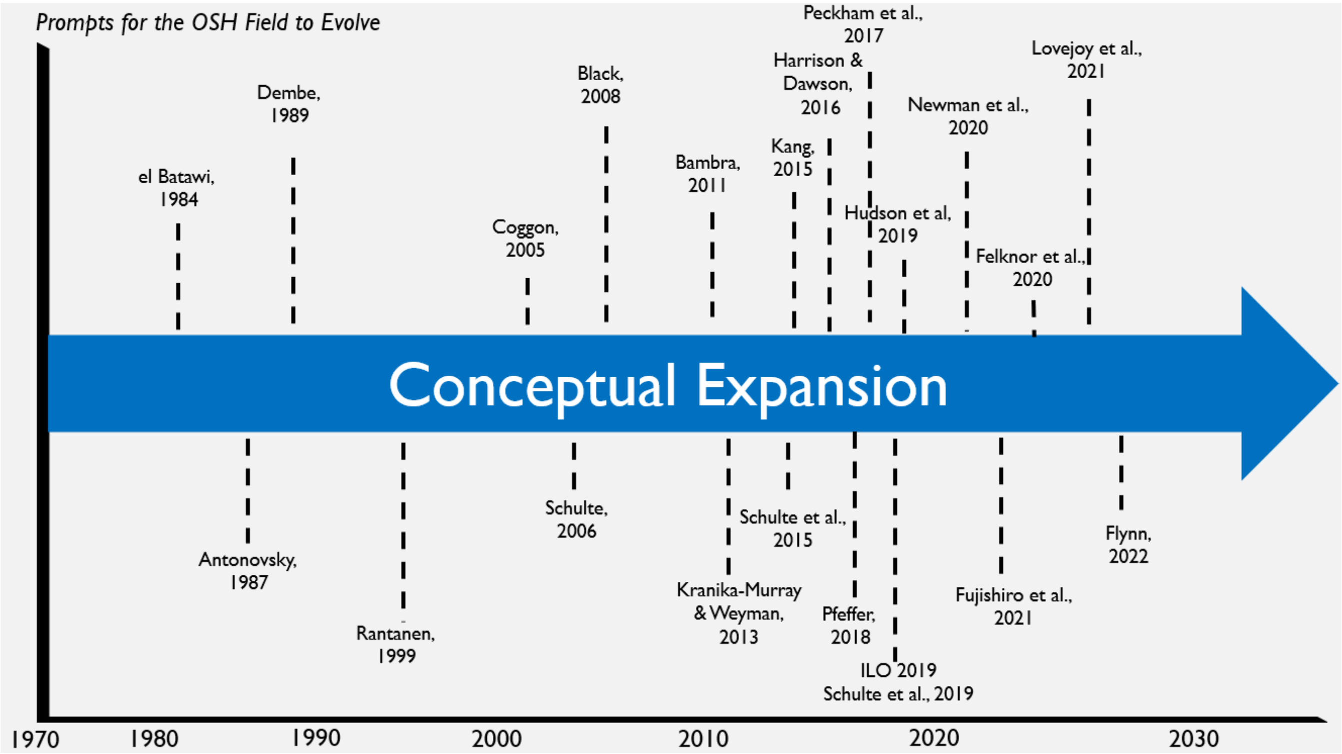

[1] The results of a horizon scan based on authors' judgement and knowledge.
[2] See Table S1 for reference numbers.

Figure S1. Literature indicating conceptual expansion of OSH[1,2].

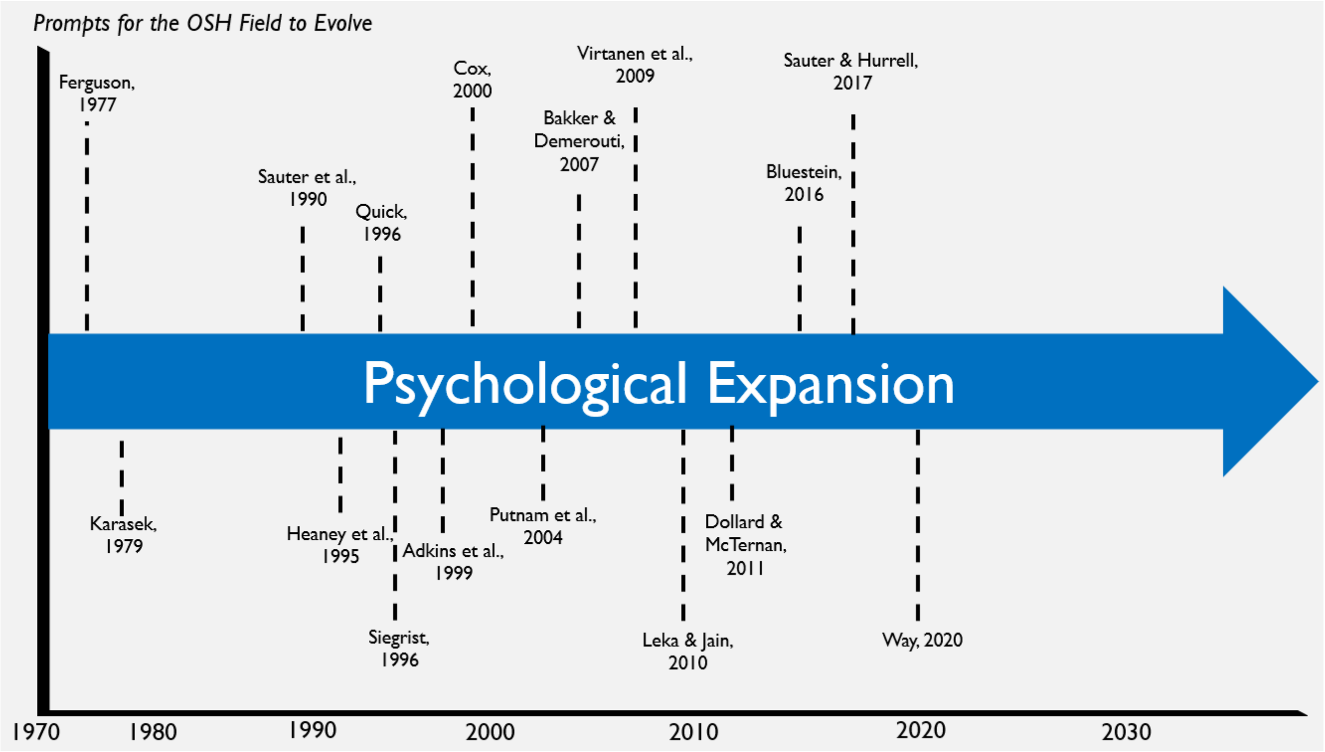

[1] The results of a horizon scan based on authors' judgement and knowledge.
[2] See Table S1 for reference numbers.

Figure S2. Literature indicating psychological expansion of OSH[1,2].

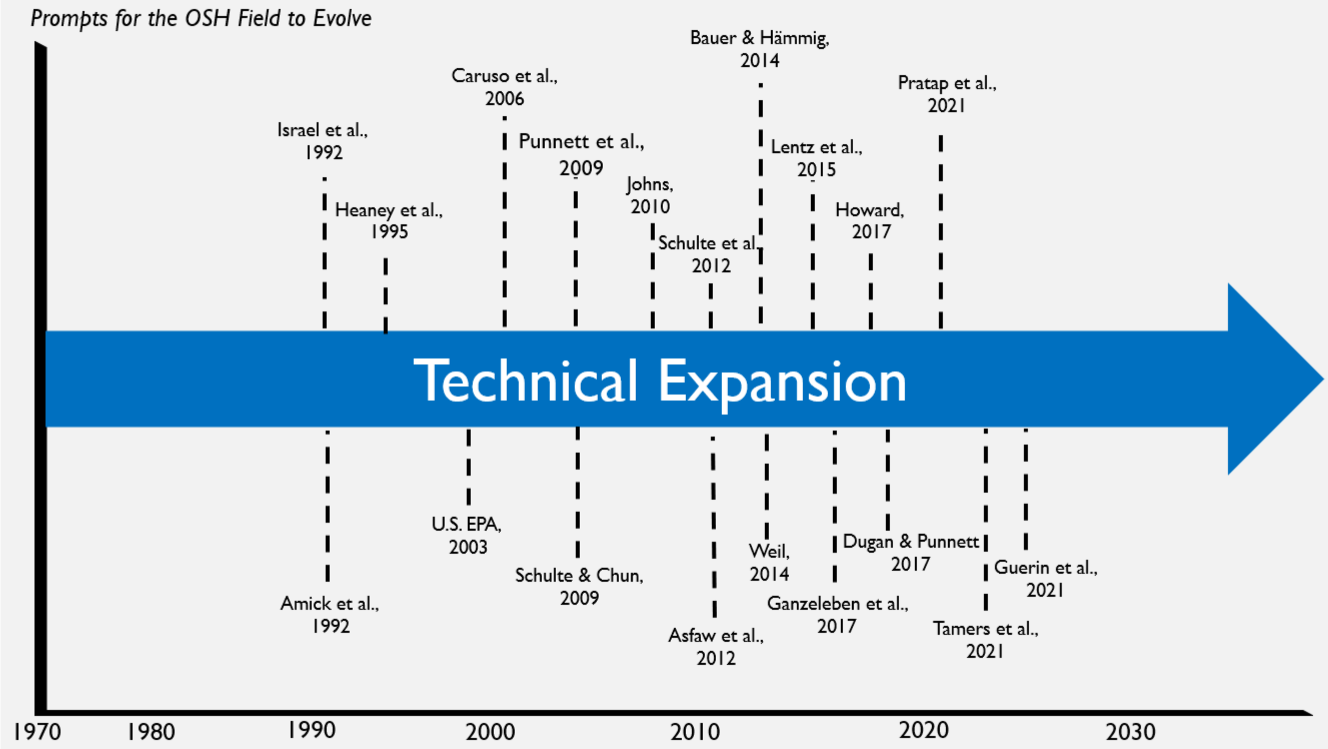

[1] The results of a horizon scan based on authors' judgement and knowledge.
[2] See Table S1 for reference numbers.

Figure S3. Literature indicating technical expansion of OSH[1,2].

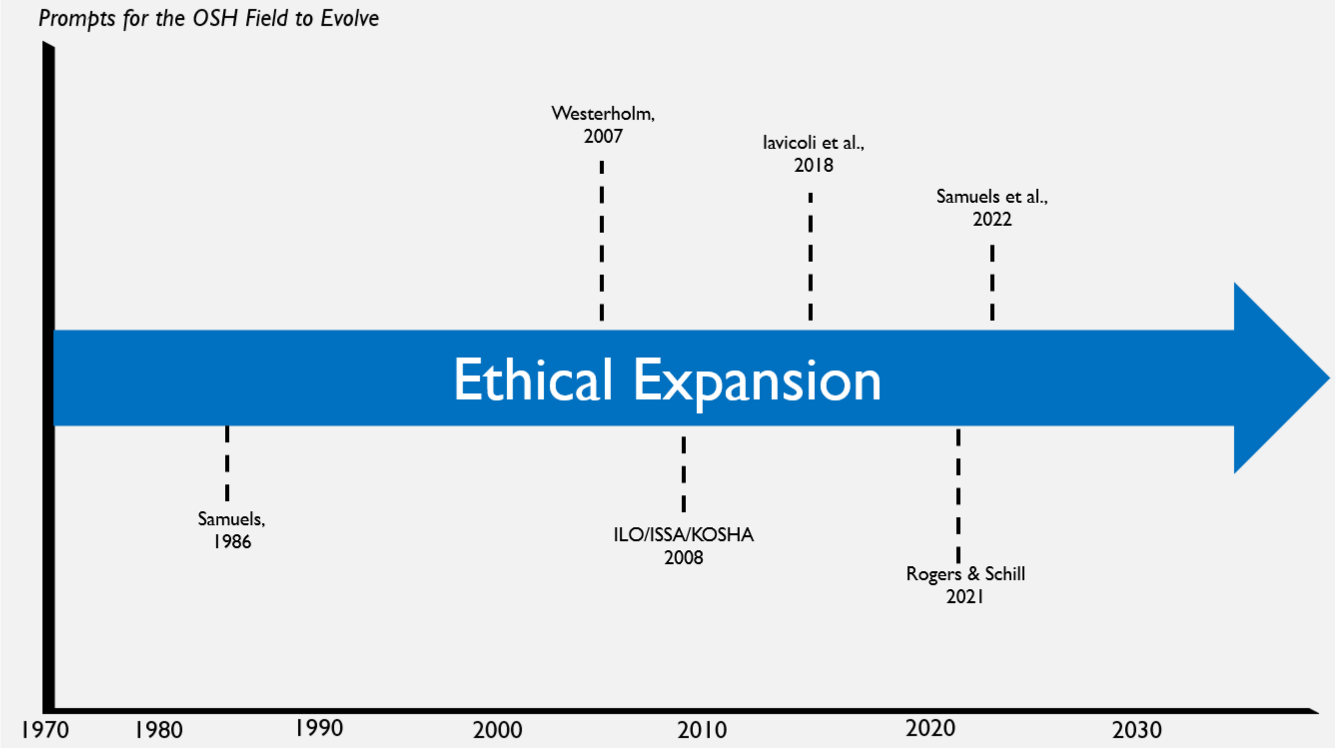

[1] The results of a horizon scan based on authors' judgement and knowledge.
[2] See Table S1 for reference numbers.

Figure S4. Literature indicating ethical expansion of OSH[1,2].
